# Migratory-derived resources induce elongated food chains through middle-up food web effects

**DOI:** 10.1186/s40462-024-00496-4

**Published:** 2024-08-20

**Authors:** Coralie Moccetti, Nicola Sperlich, Grégoire Saboret, Hanna ten Brink, Jakob Brodersen

**Affiliations:** 1 Department of Fish Ecology and Evolution, Center for Ecology, Evolution & Biogeochemistry, Eawag: Swiss Federal Institute of Aquatic Science and Technology, Kastanienbaum, 6047 Switzerland; 2 Division Aquatic Ecology & Evolution, Institute of Ecology and Evolution, University of Bern, Bern, 3012 Switzerland; 3 Institute of Biogeochemistry and Pollutant Dynamics, ETH Zürich, Universitätstrasse 16, Zürich, 8092 Switzerland; 4 Present Address: Institute for Biodiversity and Ecosystem Dynamics (IBED), University of Amsterdam, PO Box 94240, Amsterdam, 1090 GE The Netherlands; 5 Department of Coastal Systems, NIOZ Royal Netherlands Institute for Sea Research, PO Box 59, Den Burg Texel, 1790 AB The Netherlands; 6 Department of Surface Waters; Biogeochemistry, Eawag: Swiss Federal Institute of Aquatic Science and Technology, Kastanienbaum, 6047 Switzerland

**Keywords:** Arctic charr, Cannibalism, Food chain length, Growth rate, Marine-derived resources, Partial migration, Size-structure

## Abstract

**Background:**

Seasonal movements of animals often result in the transfer of large amounts of energy and nutrients across ecosystem boundaries, which may have large consequences on local food webs through various pathways. While this is known for both terrestrial- and aquatic organisms, quantitative estimates on its effects on food web structure and identification of key pathways are scarce, due to the difficulty in obtaining replication on ecosystem level with negative control, i.e. comparable systems without migration.

**Methods:**

In this study, we estimate the impact of Arctic charr *(Salvelinus alpinus)* migration on riverine ecosystem structure, by comparing multiple streams with strictly resident populations above natural migration barriers with streams below those barriers harboring partially migratory populations. We compared density estimates and size structure between above and below populations. Diet differences were examined through the analysis of stomach contents, changes in trophic position were examined by using stable isotopes. To infer growth rate of resident individuals, back-growth calculation was performed using otoliths.

**Results:**

We find higher densities of small juveniles in partially migratory populations, where juvenile Arctic charr show initially lower growth, likely due to higher intraspecific competition. After reaching a size, where they can start feeding on eggs and smaller juveniles, which are both more frequent in partially migratory populations, growth surpasses that of resident populations. Cannibalism induced by high juvenile densities occurred almost exclusively in populations with migration and represents an altered energy pathway to the food web. The presence of large cannibalistic charr feeding on smaller ones that have a similar trophic level as charr from strictly resident populations (based on stomach content) coupled with steeper δ^15^N-size regression slopes illustrate the general increase of food chain length in systems with migration.

**Conclusions:**

Our results thus suggest that the consumption of migration-derived resources may result in longer food chains through middle-up rather than bottom-up effects. Furthermore, by occupying the apex of the food chain and feeding on juvenile conspecifics, resident individuals experience reduced competition with their young counterparts, which potentially balances their fitness with migratory individuals.

**Supplementary Information:**

The online version contains supplementary material available at 10.1186/s40462-024-00496-4.

## Background

Seasonal movements of vast amounts of animals between various habitats represent one of the most remarkable phenomena in the biosphere. This collective behavior, known as migration, is globally widespread across many animal taxa and allows for opportunities to feed or breed exceeding the advantages of staying in a single habitat [1, 2]. By moving between environments, migratory individuals have potential to alter food webs through several mechanisms such as predation or competition but also through the transport of resources (i.e. nutrients and energy) acquired in the migratory habitat [3]. Substantial impacts on ecosystem productivity have been demonstrated due to nutrient inputs by migratory individuals in a diverse array of taxa, including birds [4, 5], mammals [6–8] and fish [9]. For instance, in Atlantic salmon (*Salmo salar*), the amount of nitrogen and phosphorus delivered through excretion and gametes of migratory individuals can be linked to a rise in freshwater productivity, specifically in terms of biofilm biomass [10].

Migratory animals can also represent significant prey resources for resident organisms [11–13], and their seasonally fluctuating abundances hold significant consequences for the foraging behavior of predators [14]. Temporary absence of a migratory prey can lead to a decrease in the somatic condition of resident predators [15], which have to employ different foraging strategies to mitigate those effects. As migratory prey abundance declines, predators can use alternative energy sources. For example, lions (*Panthera leo*) and cheetahs (*Acinonyx jubatus*) switch between feeding on migratory wildebeest (*Connochaetes taurinus*) and resident buffalo (*Syncerus caffer*) according to wildebeest migration [16]. Another strategy consists of consuming an unusually large amount of food resources within a short period of time, i.e. binge feeding. Binge feeding is frequently associated with prey returning or out-migrating as seen in bull trout (*Salvelinus confluentus*) foraging on sockeye salmon smolts (*Oncorhynchus nerka*) or arctic foxes (*Alopex lagopus*) feeding on eggs from migratory geese *(Chen rossii*,* Chen caerulescens*) [17, 18]. This strategy can enable the storage of excess energy, which can be utilized during periods of reduced prey availability promoting the growth of consumers [15, 19]. Resource pulses can therefore benefit resident consumers through consumption of migration-derived resources.

It is widely accepted that migratory animals are important for food web dynamics [15, 20, 21] and that resources from migratory animals can be important subsidies for organisms at higher trophic levels (e.g. [21]). However, robust studies on the subsequent effects on the food web structure are scarce. Such incorporation is imperative to study as it may lead to different food web structures than predicted solely from bottom-up nutrient enrichment scenarios, and potentially alter food chain length. Food-chain length, defined as the number of transfers of energy or nutrients from the base to the top of a food web and quantified as the maximum trophic position found in a food web [22] can influence community structure by altering the organization of trophic interactions [23]. Salmonid fish provide unique study systems to study the impacts of migratory individuals on food web dynamics. They exhibit remarkable migratory behaviors such as anadromy, where individuals that hatched in freshwater migrate to the ocean, and later return to breed in their native streams or lakes [24]. They are of massive importance for riverine ecosystems across the temperate-, sub-Arctic- and Arctic Zone in the Northern Hemisphere. Salmonids often act as keystone species [25], with large influence on food web structure [3] and nutrient dynamics [9, 26]. It is well recognized that the enrichment of streams through pulses of carcasses or eggs can increase the density and biomass of certain aquatic invertebrates [27, 28] that function as main fish prey. Additionally, the high energetic value of salmon eggs [29] has the potential to increase the growth and condition of juvenile fish that consume them [30–32]. Conversely to the beneficial aspect of migration, salmonid return to freshwater is often associated with an increased juvenile density due to the high fecundity of large migratory females producing a greater number of eggs as opposed to resident individuals [33], thereby affecting density dependent factors such as competition for resources. Juveniles may however also act as prey for large non-migratory individuals and therefore represent an additional energy pathway in the food web.

To our knowledge the changes in population structure and cascading effects on trophic dynamics associated with different pathways of marine-derived resources into coastal food webs remains unclear. To grasp the magnitude of the effects of migratory behavior on non-migratory individuals and their food webs, it is imperative to establish simple (e.g. one species present) replicate comparisons between systems with negative control (i.e. no migration). Partial migration, where some populations exhibit intra-population variation in migratory propensity [34] offers such a valuable opportunity as it allows for direct comparison between groups of the same species. Although a recent study has given indication that partial migration can have ecological effects on the density and size-structure of salmonid fish [33], a lack of independent replicates complicates the generalization of the results. This strategy also offers the opportunity to understand how different energy pathways might alter the balance between fitness in migrants and residents, which is determinant for partial migration to be maintained and has so far been unexplored. Access to marine-derived resources can be of high importance for resident individuals as it might positively impact their fitness and therefore their persistence in systems influenced by migration.

In this study, our aim is to evaluate the influence of partial migration on food web structure as well as the ecology and size-structure of non-migratory individuals from partially migratory populations. This may additionally provide details towards how migration-derived resources affect negative and positive feedbacks in the balance in partial migration. Arctic charr (*Salvelinus alpinus*) populations provide an ideal study system for this investigation as they are known to exhibit several life histories by being either partially migratory or strictly residents [24]. Migratory charr are iteroparous and migrate to the ocean for feeding and rearing, and then return to streams to breed and overwinter whilst resident charr remain in freshwater throughout their life. Arctic charr populations occur in numerous drainages both below- and above waterfalls in Southern Greenland. These natural barriers prevent upstream movements from sections below, resulting in strict resident (above) and partial migratory (below) populations (i.e. comprising both resident and migratory individuals). In addition, in these habitats Arctic charr are the only fish species present, resulting in a simple ecosystem, which allows us to understand functional pathways.

By comparing juveniles and resident individuals from above- and below-barrier reaches in seven different rivers, we expect that partial migration in Arctic charr has an effect on the ecology and size structure of the resident individuals living below waterfalls. We predict that on the population level, partially migratory populations will, in comparison to fully resident populations, exhibit (1) a higher density of juveniles due to the high fecundity of returning migratory females (2) larger resident individuals than strictly resident populations due to increased cannibalism on eggs and juveniles, inducing high growth (3) steeper δ^15^N-size slopes. In addition, we predict that on the individual level, resident individuals from partially migratory populations will in comparison to individuals from resident populations (1) grow slower early in life due to high competition with conspecifics (2) grow better after reaching a certain size that allows them to benefit from marine-derived resources input through cannibalism.

## Methods

### Study system

**Fig. 1:**
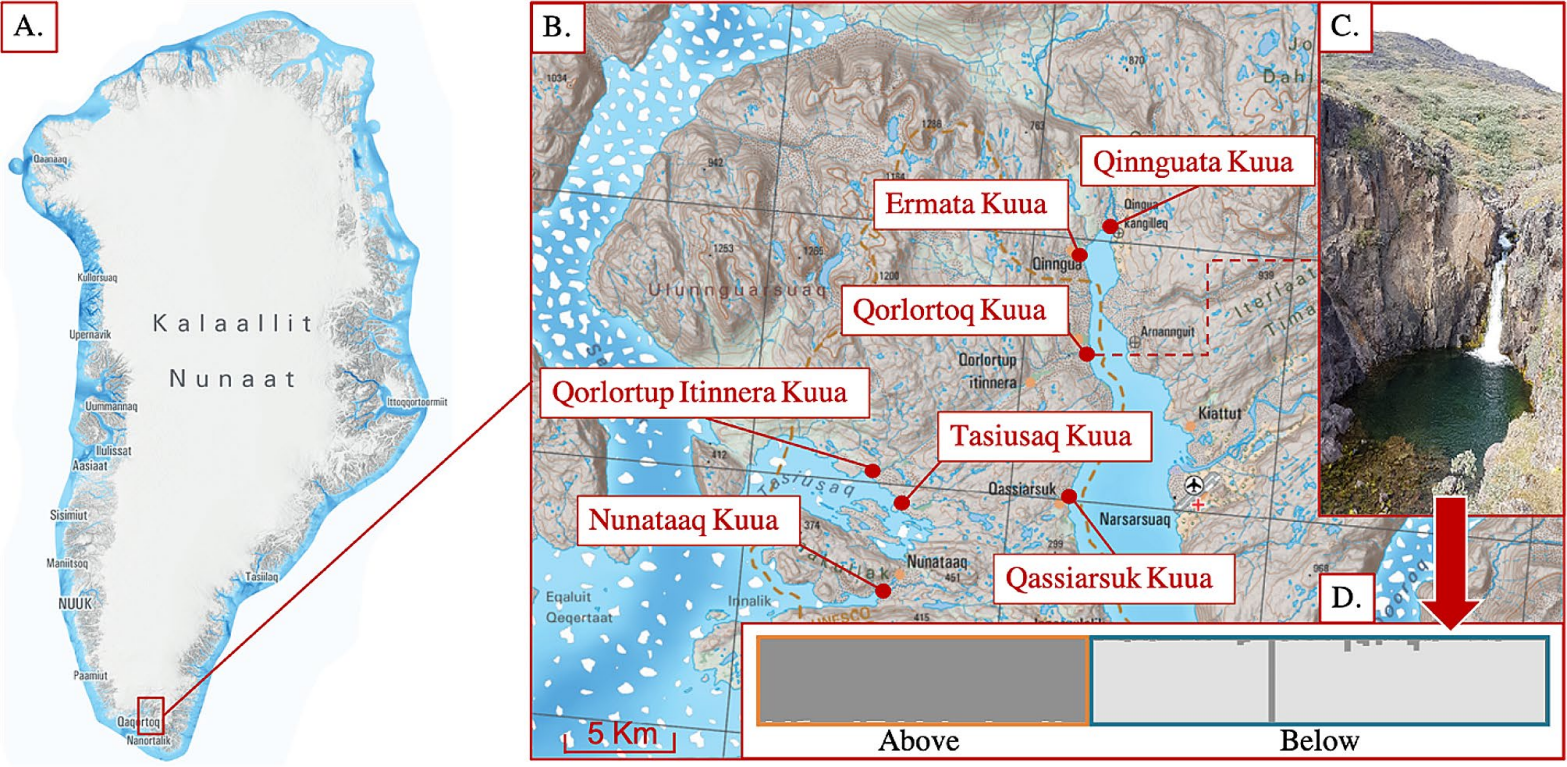
**(A)** Greenland (Kalaallit Nunaat [35] with **(B)** sampled streams that have been colonized by Arctic charr (*Salvelinus alpinus*). **(C)** In each location resident populations inhabit streams above waterfalls that prevent upstream movements from sections below that are occupied by partially migratory populations. **(D)** Plot of genetic assignment of an example of two of the populations from one stream living above (*n* = 52) and below (*n* = 73) waterfalls that are reproductively isolated, as obtained in the hierarchical STRUCTURE analysis

This study was conducted in southern Greenland (61°12’0"N, 45°32’0"W; Fig. 1), where numerous streams have been colonized by Arctic charr (*Salvelinus alpinus*). We sampled seven streams as study systems due to their relative simplicity (no other fish species present), low anthropogenic influence and ease of access on foot from unpaved roads. We included all streams in the study area (Erik the Red’s land on the Narsaq Peninsula), which had anadromous charr below a barrier large enough to restrict upstream migration and which had charr populations living above it. (Additional file 1: Table S1 for details). In each stream, we sampled a strictly resident charr population inhabiting a section above natural barriers (e.g. waterfalls) that prevent upstream movements from sections below where we sampled a partially migratory population. Migratory individuals refer solely to a subset of individuals from populations living below the barriers that migrate and have been characterized by their migratory phenotype in the field (e.g., silver coloration, relative head and fin size, body shape, adult stage, see 42). Resident individuals below the barriers refer to the non-migratory individuals that have been characterized by their resident phenotype in the field (e.g., brown coloration, adult stage). All individuals that have not been sexed because their gonads were not visible were characterized as juveniles. The study system allows us to assess the ecological effects of partial migration on resident individuals by comparing above- and below barrier sections.

To confirm that upstream sections are not accessible to migratory individuals, genetic structure analysis using nine microsatellites were performed between above and below waterfall populations. Partially migratory populations are defined by non-significant Fst values (*p* > 0.05) between resident and migratory individuals.

### Biotic and abiotic variables

In 2021 abiotic and biotic variables were recorded where the fish were collected. Aquatic invertebrate abundance in each stream above and below barriers was assessed except for one stream (Qinnguata Kuua below). A hand net with 1 mm mesh net bag was set at the bottom of the river, a researcher kicked ten times backwards in the sediment 50 cm upstream the net to agitate the sediment and stones. After removing big debris, the net content was emptied in 200mL vials, this process was repeated five times moving upstream. Aquatic invertebrates were classified (chironomidae larvae, chironomidae pupae, simuliidae larvae, trichoptera larvae, trichoptera pupae), counted and a Poisson-distributed Generalized Linear Mixed Effects Models (GLMM) with a log link function using the ‘glmmTMB’ package in R [36] was performed to compare their abundance between above and below barrier stretches; aquatic invertebrate count was assigned as response variable, barrier (above/below) as fixed factor and stream as random factor. Overdispersion in the residuals and zero-inflation were tested using the DHARMa package [37].

Average stream width was measured with ten evenly spaced measurements in six out of the seven streams sampled as Qinnguata Kuua was too wide and the flow too high to walk-in. A Linear Mixed Model (LMM) was performed to compare stream width between above and below barrier stretches; stream width was assigned as response variable, barrier (above/below) as fixed factor and stream as random factor. Normality assumption was tested with a Kolmogorov-Smirnov test. A Levene’s test was used to test if the residuals of the model differed significantly across the above and below barrier stretches within streams.

### Sample collection, density estimates and size structure

Fish sampling took place across three years from early July to late August (2018, 2019) and from early September to early October (2021). In 2018 and 2019 we sampled resident fish by qualitative electrofishing (i.e., targeting juveniles and adults) until a sufficient number of individuals representing different size-classes were caught (minimum of 20 individuals per stream) for size structure estimation. To estimate fish density, we did semi-quantitative electrofishing using a DC backpack electrofishing device (i.e., sampling all individuals in the stretch during one fishing effort) in 2021 in heterogeneous stretches that represent all the possible habitats used by the fish. Six streams were sampled out of seven for this analysis, as one stream (Qinnguata Kuua) was too large and turbid to sample quantitatively. We estimated the surface area of each stretch by multiplying the length by average width. A piece of the right pectoral fin and of dorsal muscle tissue were taken and stored in 100% ethanol for DNA and stable isotope analysis, respectively. Fish were sexed, standard size (SL, (mm)) was recorded, and specimens were stored in formaldehyde (4%) for three months before being transferred in 70% ethanol. The same methods were applied to sample migratory individuals that were solely used for genetic analysis and egg density estimation in that study. Stomachs of resident individuals were extracted and the content was counted and classified by order or family when possible.

Male, female and juvenile expected densities were compared between populations living above and below barriers. Fish densities were modeled by fitting a Poisson-distributed Generalized Linear Mixed Effects Models (GLMM) with a log link function. By using count of fish as the response variable and including log of sampling area as offset, the models predicted the number of fish while adjusting for differences in sampling area. Stream was used as a random factor for each model. Aquatic invertebrate abundance and stream width were used as fixed factors for each model as well as their interactions with the barrier, overdispersion in the residuals and zero-inflation were tested.

To visualize the distribution of fish lengths above and below barriers a Kernel density plot was used on pooled streams including 482 fish above and 699 fish below the barriers. A Kolmogorov-Smirnov test was applied to compare if the standard length distributions were similar between above and below populations.

We fitted a quantile regression model at 80th percentile representing the upper tail of the standard length distribution and compared the coefficients for the different groups of the barrier variable in order to assess whether largest fish were found below the barrier as compared to above. The model estimated the relationship between the predictor variable barrier and the response variable standard length for the upper tail of the data distribution (top 20%).

To visualize the proportion of resident females sampled in all years (*n* = 92 above, *n* = 36 below) between populations living above and below barriers a mixed-effects logistic regression model was fitted with female presence/absence as response variable, barrier as fixed effect and stream as random factor. Normality assumption of the model was tested with a Kolmogorov-Smirnov test. A Levene’s test was used to test if the residuals of the models differed significantly across the above and below barrier populations within streams.

### Microsatellite genotyping and genetic data analysis

A total of 1148 individuals including juveniles, residents and migratory individuals were genotyped at nine microsatellite loci: OMM1228, OMM5151, OMM1329, OMM1236, OMM1211, OMM5146, OMM1302, BX890355 [38–40] and Ssa100 (alias BHMS321) (Hoyheim, B. unpublished, primer sequences in [41] as previously done in a study on Greenlandic charr [42]. DNA was extracted from fin clips using 150 µl Chelex ^®^ 100 (Bio-RAD) in 5% concentration diluted in ultra-pure water with 10 µl of TE buffer and 5 µl of proteinase K (10 mg/ml) added. Markers were amplified using a PCR multiplex containing 2.5 µl Qiagen PCR Multiplex Kit (Qiagen, Basel, Switzerland), 1.75 µl DNAse- free water, 0.135 µl of primer mix, and 0.75 µl of extracted DNA per reaction. PCR consisted of an initial denaturation step of 15 min at 94 °C followed by 30 cycles of 30s at 94 °C, 90s at 55 °C and 90s at 72 °C, with a final elongation step of 30 min at 60 °C. The PCR product was diluted with 25 µl ultra-pure water and a denaturation step at 92 °C was applied for 2 min. Denaturized product was loaded on the Applied System 3130xl Genetic Analyzer for fragment analysis with 0.15 µl LIZ600 size standard and 18 µl ABI-HiDi per sample. Peaks were scored with the software Genemapper v. 4.0. We used a hierarchical approach with the software STRUCTURE [43] to assess the genetic structure of each stream. We ran above and below barriers populations together with K = 1–4, with 10 replicates, 50000 burn-in and Markov Chain Monte Carlo algorithm steps each, applying the admixture model for correlated allele frequencies. Structure Harvester [44], which implements the Evanno method [44], was used to identify the optimal likelihood of K. GENALEX software was used to convert files from STRUCTURE to GENODIVE format [45]. Pairwise multilocus FST (1000 permutations) was calculated between above and below populations for each stream with the software GENODIVE [46].

### Diet analysis

Charr larger than 200 mm were removed from the stomach content and stable isotope analysis in order to compare fish of similar size range. Difference in average standard length between populations comprising juveniles and resident fish living above and below barriers was tested using a non parametric Kruskal-Wallis test followed by pairwise comparisons using Wilcoxon rank sum test with Bonferroni correction. Standard length was assigned as response variable and barrier (above/below) as predictor.

In order to have a good representation of diet in various size ranges, we randomly retrieved stomach contents from 538 individuals sampled in 2018 and 2019 and representing 88% of our total catch above barrier and 66% below barrier. In addition, we retrieved randomly 50 stomach contents from individuals caught in fall 2021 to record for the presence or absence of egg cannibalism.

We identified prey from stomachs to family level and subsequently pooled them in two main categories according to their general quality as food item (aquatic invertebrates (aquatic macroinvertebrate larvae and adults re-entering aquatic systems primarily through dispersal flights) and terrestrial prey (entering from land-based habitats)) (Additional file 1: Table S2 for details of prey). A Generalized Additive Model (GAM) with a tweedie distribution was applied for the aquatic invertebrate category found in 277 fish above and 261 fish below the barriers. We used the count of prey as response variable, and for the explanatory variables the barrier (above/below), the standard length of the fish and their interaction. In addition, we included year and stream as random factors. The fit of the model was visualized using the *gam.check* function from the mgcv [47] package and the R package ggplot2 [48]. Terrestrial prey were found in few of the stomachs (*n* = 7 below, *n* = 32 above) so a Generalized Additive Model (GAM) could not be fitted. To predict the presence/absence of terrestrial prey in the stomach content of residents and to test how the presence/absence depends on the size and location of the fish, we fitted a logistic regression with the presence/absence of the terrestrial prey as the response variable and the barrier, standard length, and their interaction as the explanatory variables. A likelihood ratio test using chi-square distribution was performed on the model to compare the probability of feeding on terrestrial prey between populations living above and below barriers.

To predict the presence/absence of charr (sampled in summer 2018 and 2019, *n* = 278 above and 275 below barriers) and eggs (sampled in fall 2021, *n* = 14 above and 36 below barriers) in the stomach content of residents and to test how the presence/absence depends on the size and location of the fish, we fitted a logistic regression for each prey type with the presence/absence of the specific prey as the response variable and the barrier, standard length, and their interaction as the explanatory variables. A likelihood ratio test using chi-square distribution was performed on both models to compare the probability of cannibalism between populations living above and below barriers.

We used published data on fecundity (egg number) of Arctic charr at different sizes [49, 50] and fitted a linear model on those data to predict the number of eggs produced by each caught female in the semi-quantitative survey according to their standard length. The total number of eggs per stretch (above/below within stream) was then divided by fished area to calculate an estimate of egg density in a reach. A non parametric Kruskal-Wallis test followed by pairwise comparisons using Wilcoxon rank sum test with Bonferroni correction were used to statistically test for differences in egg density between above and below barriers.

We performed a linear regression analysis to assess the relationship between the standard length of the juvenile charr found in the stomach of resident charr and the size of their consumers (Additional file 2: Figure S1). Due to prey degradation, only 19 prey fish from the 14 cannibalistic charr could be measured.

**Fig. 2:**
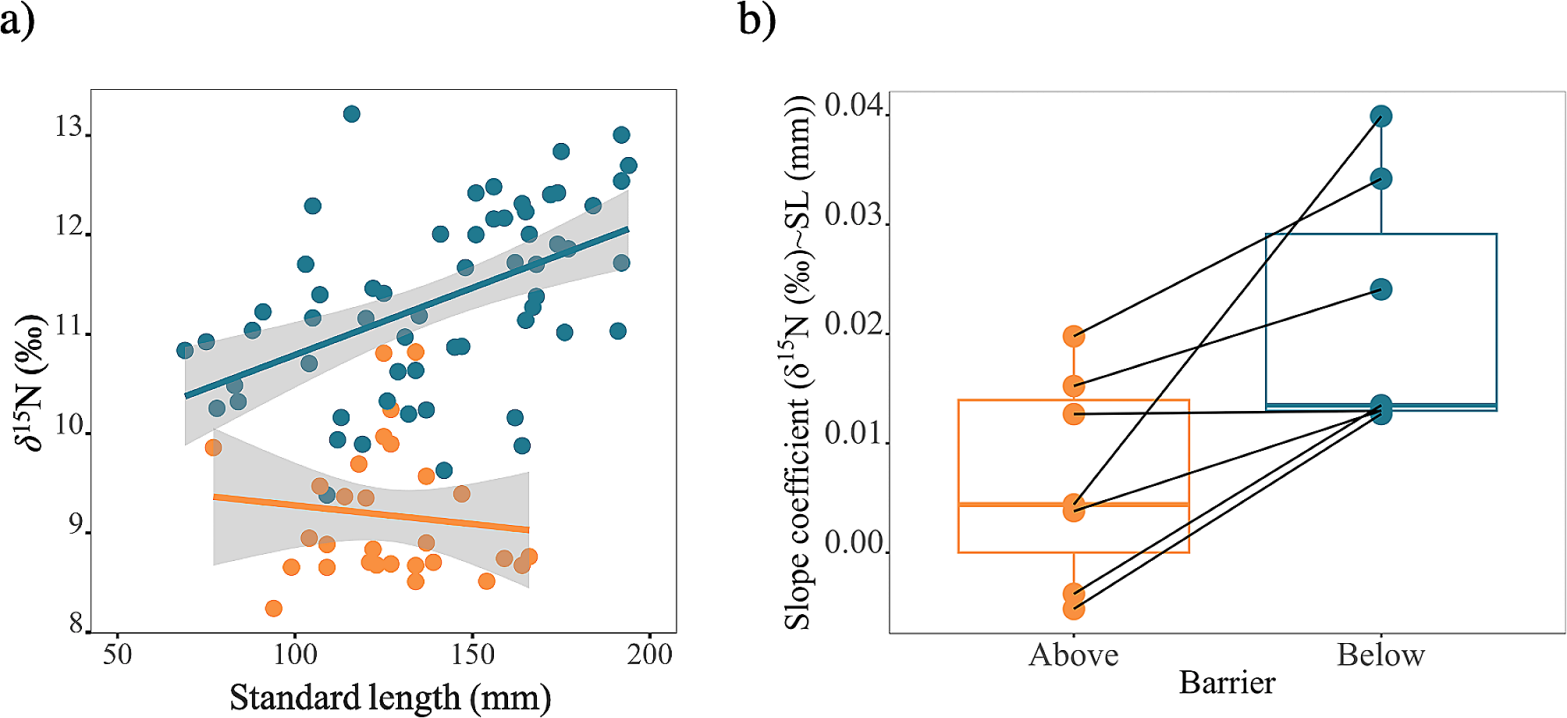
**(a)** Illustration of the relationship between bulk stable isotopes of nitrogen (y-axis) and standard length of Arctic charr (x-axis) for a single stream as example between above and below populations. **(b)** Slope coefficients extracted from linear regressions of δ^15^N against standard length within above and below populations for each stream

We performed stable isotope analysis for 220 randomly sampled individuals above the barriers representing 70% of our total catch above waterfalls and 264 below the barriers representing 66% of our total catch below waterfalls. In the laboratory, the ethanol preserved dorsal muscle tissue samples were dried in aluminum foil in a standard laboratory drying oven for 48 h and subsequently crushed to a fine homogenate powder using tungsten beads (3 min at 30 Hz). Ethanol has little effect on the isotopic composition of nitrogen [51]. Nitrogen stable isotope values (δ^15^N) were determined at the Eawag department of Surface Waters-Research and Management using approximately 1 mg of dried homogenate processed with Vario PYRO CUBE CN elemental analyser (Elementar, Langenselbold, Germany) connected to an isotope ratio mass spectrometer (IRMS, Elementar, Langenselbold, Germany). Urea and acetanilide standards with certified values (Schimmelmann, Indiana University) were ran every tenth sample. Results are reported in standard delta (δ) notation as parts per thousand (per mil, ‰) relative to the international standards for nitrogen [52]. Baseline signatures between streams could not be corrected, therefore we did not use absolute values of δ^15^N but we extracted slope coefficients from linear regressions of δ^15^N against standard length within above and below barriers populations, for each stream separately (Fig. 2a; see Additional file 2: Figure S2 for all streams). Slope coefficients can inform us about a potential change of δ^15^N with individual size, reflecting changes in the trophic structure of the community. A linear mixed model was fitted to test for differences in slope coefficients between above and below barrier populations. Slope coefficient was assigned as response variable, barrier (above/below) as fixed effect, stream and year were assigned as random factors. Normality assumption of the model was tested with a Kolmogorov-Smirnov test. A Levene’s test was used to test if the residuals of the model differ significantly across the above and below barrier populations within streams.

### Back-growth calculation

Pairs of otoliths were extracted from 128 fish above and 149 fish below the barriers, cleaned with water and dried (see Additional file 1: Table S3 for sampling details). The samples were glued to a glass slide, sulcus side down, using CrystalbondTM 509, and polished by hand with 20 μm lapping film until all growth zones were revealed and a flat surface was obtained. Otoliths were then immersed in water and photographed using a stereo-microscope and camera apparatus (LEICA M205 A). Wide clear summer growth zones followed by narrow dark winter zones, combined called annuli, represent yearly growth patterns in an otolith [53]. The distance from the nucleus to each individual annulus was measured using Fiji software [54]. Measurements were taken along the longest ventral axis, from the center of the nucleus to each individual annulus at the end of the winter dark zone (Additional file 2: Figure S3). These measurements provided otolith size-at-age data. Due to strong correlation between the size of the otolith and body size of fish, back-calculation models assume that there is a linear relationship between the growth of the otolith (increment width) and the somatic growth, usually length, of the fish. We used the linear biological intercept model developed by Campana (BCM) [53, 55] to estimate size at age of resident fish. The length of an individual at age *i* is calculated as:

1 $${L_i} = {L_{cpt}} + \left( {{R_i}--{R_{cpt}}} \right)*{\text{ }}\left( {{L_{cpt}}--L0h} \right)*{\left( {{R_{cpt}}--R0h} \right)^{ - 1}}.$$

In this equation, L_*i*_ is the fish length (mm) at age *i*, L_cpt_ the length at capture (mm), R_*i*_ is the otolith radius at age *i* (mm), Rcpt is the otolith radius at capture (mm), R0h the otolith radius at hatch (mm), and L0h the length at hatch (mm). Data of a partially migratory population of Arctic charr *(Salvelinus alpinus)* sampled in Iceland (*Fljótaá)* [56] was taken as reference for L0h and estimated as 14.8 mm for resident individuals of both upstream and downstream populations. For this study, we assume that resident individuals above and below barriers have similar length at hatch.

Following analysis were done on the first 6 years of life as sample size decreased drastically after that age. A linear mixed model was fitted to compare growth between populations living above and below waterfalls. The model explained growth at age *n* + 1((estimated standard length at age *n* + 1) – (estimated standard length at age n)) as a function of the relative position to the barrier (upstream vs. downstream), the estimated standard length of the fish at age n and their interaction. This model included both stream and individual as random intercepts.

A random slope model was fitted to compare the estimated standard length at age between populations living above and below waterfalls. The model explained estimated standard length as a function of the relative position to the barrier (upstream vs. downstream), the age of the fish and their interaction. This model included both stream and individual as random intercepts, a random slope for the variable age across the different streams was included as well.

All analyses were conducted in the software R Version 2023.03.1 + 446 [57] with the packages reshape2 [58], lme4 [59], DHARMa [37], quantreg [60], ggplot2 [48], car [61], mgcv [47], glmmTMB [36] and dplyr [62].

## Results

### Biotic and abiotic factors

The abundance of aquatic invertebrates observed in 2021 was not significantly different between above and below stretches (Additional file 1: Table S4, GLMM: IRR = 1.02, *p* = 0.634; Additional file 2: Figure S4).

The stream widths measured in 2021 were not significantly different between above and below stretches (Additional file 1: Table S5, LMM: df = 5, *p* = 0.166; Additional file 2: Figure S5).

As those biotic and abiotic variables were recorded at one point in time (2021) and in one section of each stream we included them as fixed factors in the analysis of density estimates of the same year and place.

### Density estimates and population size structure

**Fig. 3:**
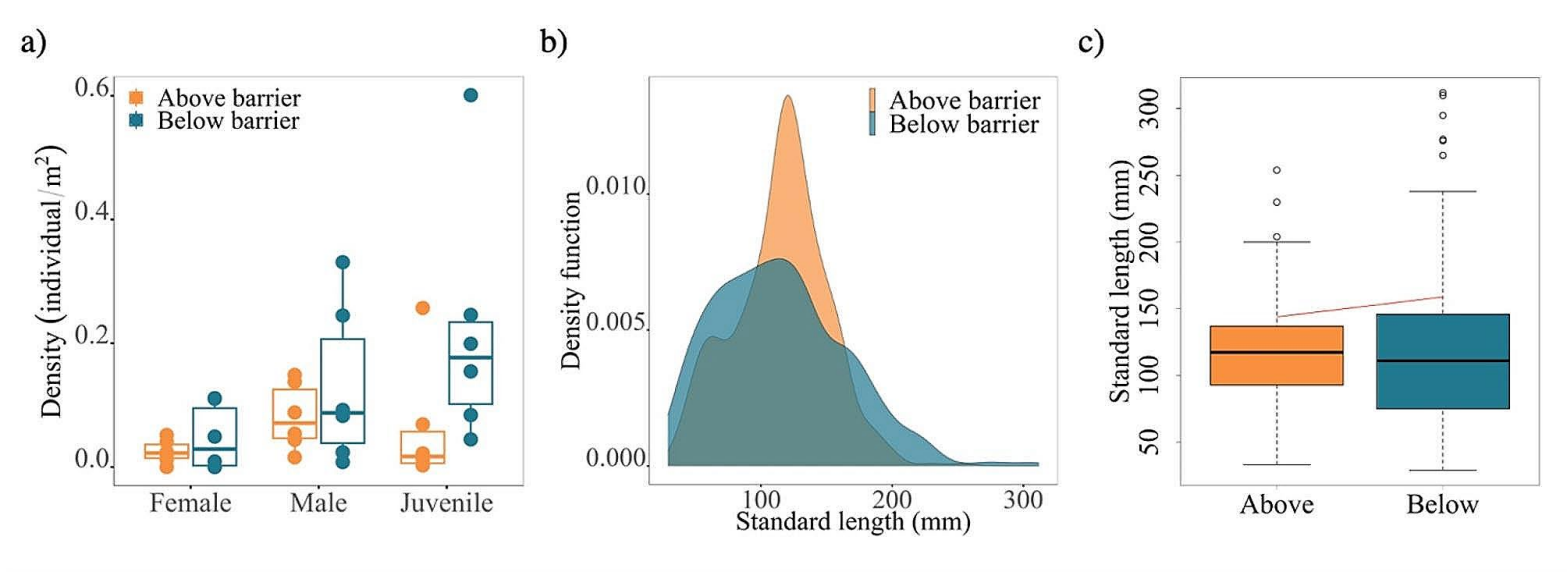
Comparisons of populations living above (juveniles, residents) and below barriers (juveniles, residents) with **(a)** density estimates of charr caught by semi-quantitative fishing in 2021, **(b)** Kernel density plot of all charr caught in 2018, 2019, and 2021 and **(c)** estimated conditional quantiles (80th percentile) of standard length for above and below barrier populations of all charr caught in 2018, 2019, and 2021. The red line represents the prediction of the 80% percentile as obtained from the quantile regression

Expected density of juveniles (immature individuals) was higher below barriers compared to above barriers (Fig. 3a; Additional file 1: Table S6, GLMM: IRR = 10.40, *p* = 0.001). Expected density of juveniles (immature individuals) was higher with increased aquatic invertebrate abundance (Additional file 1: Table S6, GLMM: IRR = 1.03, *p* < 0.001) but no significant interaction was found between barrier and aquatic invertebrate abundance (Additional file 1: Table S6, GLMM: IRR = 1.00, *p* = 0.433). Stream width had no significant effect on juvenile density (Additional file 1: Table S6, GLMM: IRR = 1.38, *p* = 0.153). No significant difference was found in male expected density between above and below barriers populations (Fig. 3a; Additional file 1: Table S7, GLMM: IRR = 1.41, *p* = 0.547). Stream width (Additional file 1: Table S7, GLMM: IRR = 0.81, *p* = 0.062) and aquatic invertebrate abundance (Additional file 1: Table S7, GLMM: IRR = 1, *p* = 0.891) had no significant effects on male expected density. Expected density of females was higher below barriers compared to above barriers (Fig. 3a; Additional file 1: Table S8, GLMM: IRR = 23.76, *p* = 0.003) and a significant interaction was found between barrier and stream width (Additional file 1: Table S8, GLMM: IRR = 0.55, *p* < 0.001). Aquatic invertebrate abundance (Additional file 1: Table S8, GLMM: IRR = 1, *p* = 0.151) had no significant effect on female expected density.

Strictly resident populations (above barriers) displayed a different distribution of standard length from the residents of partially migratory populations (below barriers) (Fig. 3b; K-S test: D = 0.133, *p* < 0.001, see Additional file 2: Figure S6 for all streams). The 80th percentile model revealed a statistically significant positive effect of below vs. above a barrier on fish size, indicating that resident charr found below the barriers are significantly larger at the upper end of the standard length distribution (Fig. 3c; see Additional file 1: Table S9, *p* < 0.01).

The proportion of resident females below barriers was estimated to be 66% lower compared to above barriers (Additional file 1: Table S10, MELR: OR = 0.34, *p* < 0.001; Additional file 2: Figure S7).

### Population genetics

Reproductive isolation was found between above and below barriers in each of the rivers (Fig. 1.D; Additional file 1: Table S11, Fst *p*-values < 0.001). Structure plots of all streams can be found in supplementary materials (Additional file 2: Figure S8).

### Diet

#### Stomach contents

Populations comprising juveniles and resident individuals sampled for stomach content analyses did not differ in average standard length between above and below barriers (K-W: X^2^ = 0.076, df = 1, pairwise comparisons using Wilcoxon rank test and bonferroni correction: p-value = 0.78).

**Fig. 4:**
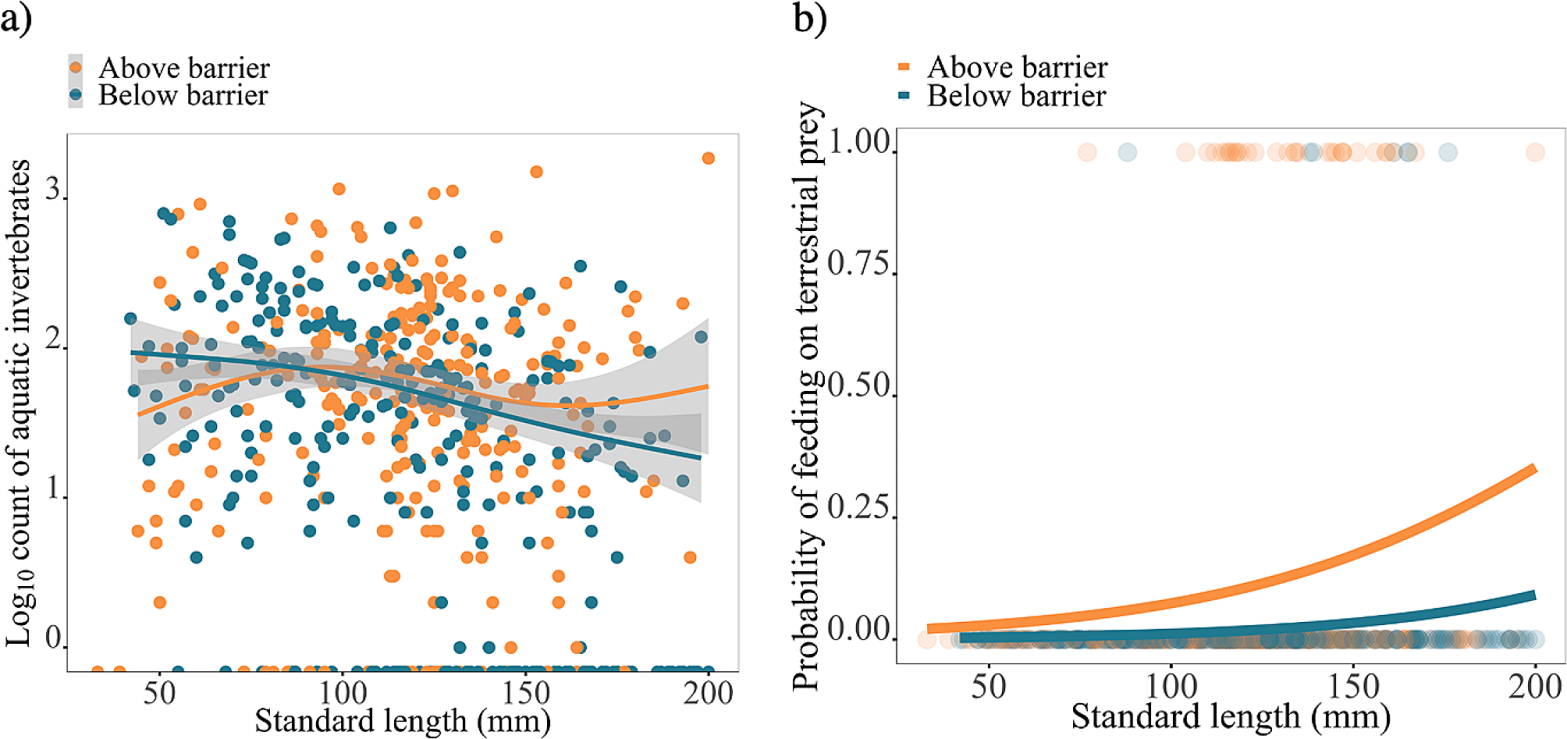
**(a)** Log_10_ of aquatic invertebrate intake found in the stomachs of resident arctic charr in relation to their size. Response curves represent predicted log_10_ count generated from a generalized additive model (GAM). **(b)** Logistic regression (GLM) showing the probability of charr to feed on terrestrial prey according to standard length between above and below barrier populations

In general, resident charr living below barriers consumed fewer aquatic invertebrates than charr living above barriers (Fig. 4a; Table 1, GAM: F_1_ = 11.602, *p* < 0.001). Aquatic invertebrate intake varies with fish size (Fig. 4a; Table 1, GAM: F_1_ = 9.189, *p* = 0.002) and the effect of standard length was stronger below barrier (Fig. 4a; Table 1, GAM: F_1.003_=20.860, *p* < 0.001) than above barrier (Fig. 4a; Table 1, GAM: F_4.282_ =3.423, *p* = 0.004). As resident charr below barriers get larger they showed a higher decrease in their aquatic invertebrate intake than residents charr living above barriers. Figures of all streams can be found in supplementary material (Additional file 2: Figure S9). Stream and year, assigned as random factors in our analyses were significant to aquatic invertebrate intake (Table 1; stream: GAM: F_5.493_ =18.697, *p* < 0.001, year: GAM: F_1.957_ =28.890, *p* < 0.001) indicating variations in the responses among streams and years.

**Table 1:** Coefficient estimates fitting a Generalized Additive Model applied to aquatic invertebrates found in the stomachs of charr from different sizes. The term “:” indicates interaction. Year and stream were assigned as random factors. We show the estimate and standard error, as well as the t- value and resulting *p*-value using a two-sided Wald test. Abbreviations: Appr. sign: approximate significance, Effective Df: effective degrees of freedom, Reference Df: reference degrees of freedom

Aquatic invertebrate intake				
**Parametric coefficients**	**Estimate**	**Standard error**	**t value**	**p-value**
Barrier (below)	-0.371	0.109	-3.406	< 0.001
Standard length	0.029	0.009	3.031	0.002
**Appr. sign of smooth terms**	**Effective Df**	**Reference Df**	**F**	**p-value**
Standard length : Above barrier	4.282	5.294	3.423	0.004
Standard length : Below barrier	1.003	1.005	20.860	< 0.001
Stream	5.493	6	18.697	< 0.001
Year	1.957	1	28.890	< 0.001

Terrestrial prey intake was more common above the barrier (Fig. 4b; Additional file 1: Table S12, LRT: X^2^ = 19.305, df = 1, *p* < 0.001), and this incidence increased significantly with standard length (Additional file 1: Table S12, LRT: X^2^ = 13.430, df = 1, *p* < 0.001)). Figures of all streams can be found in supplementary material (Additional file 2: Figure S10).

**Fig. 5:**
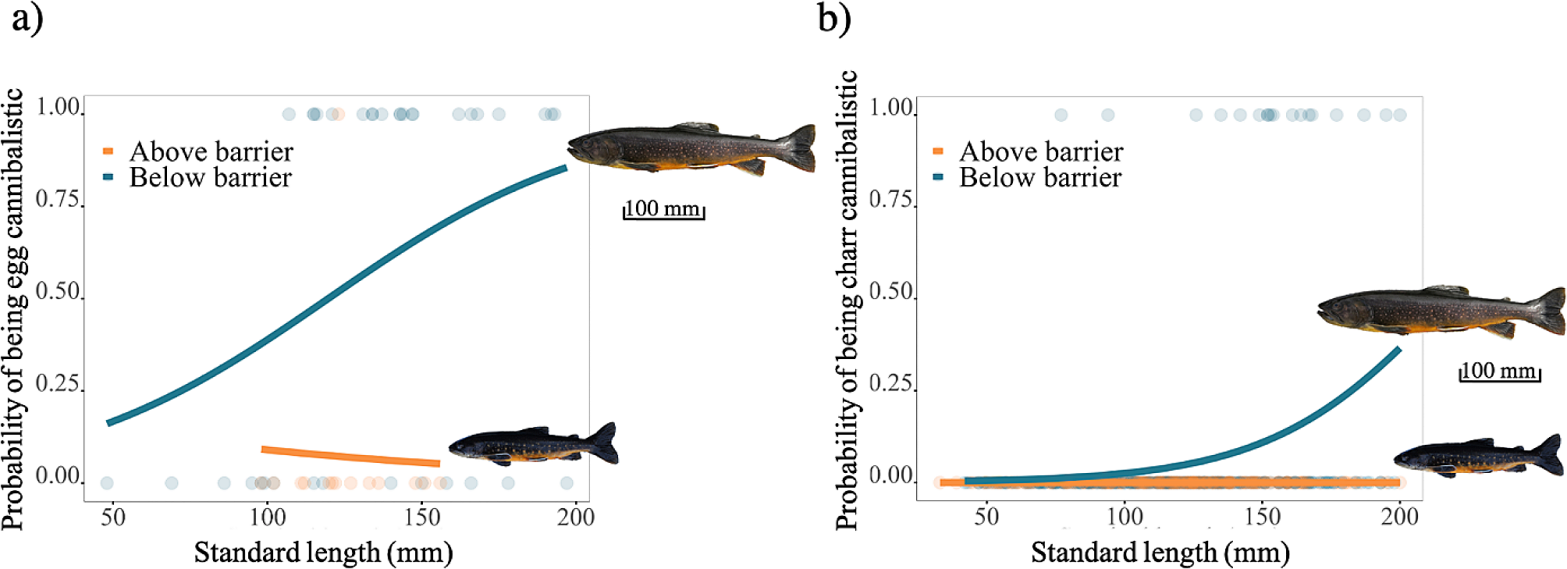
Logistic regression (GLM) showing the probability of charr to feed on **(a)** charr egg (Year 2021) and **(b)** juvenile charr (Year 2018 and 2019) according to standard length between populations of above and below waterfalls. Figures **(a)** and **(b)** show illustrations of an example of a resident cannibalistic phenotype below the barrier and a non-cannibalistic charr phenotype above the barrier with associated scale (100 mm) for reference

Egg cannibalism was more common below the barrier (Fig. 5a; Additional file 2: Figure S11 for all streams; Additional file 1: Table S13, LRT: X^2^ = 12.486, df = 1, *p* < 0.001), and this incidence increased significantly with standard length (Additional file 1: Table S13, LRT: X^2^ = 4.559, df = 1, *p* = 0.032). General cannibalism was also more common below than above the barriers (Fig. 5b; Additional file 2: Figure S12 for all streams; Additional file 1: Table S14, LRT: X^2^ = 26.702, *p* < 0.001) and this incidence increased with size (Fig. 5b; Additional file 1: Table S14, LRT: X^2^ = 19.323, *p* < 0.001). Newly hatched larvae (< 30 mm) were consumed by charr from 136 mm whilst larger juveniles were consumed by charr from 150 mm (Additional file 2: Figure S1).

#### Egg density

**Fig. 6:**
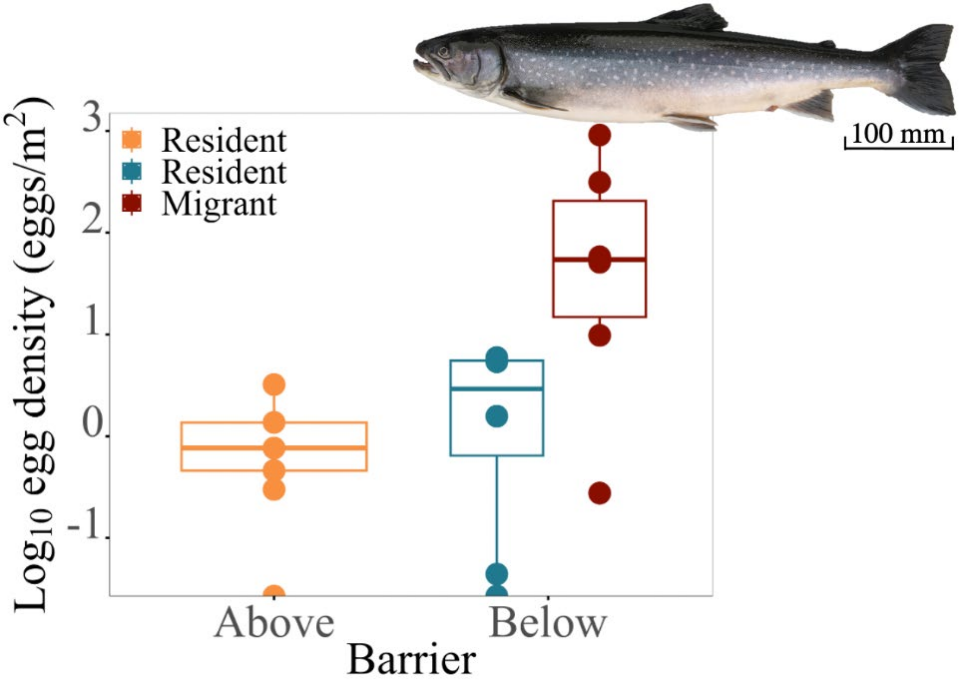
Average egg density (Log_10_) of migratory and resident charr above and below barriers. The figure shows an illustration of an example of a migratory charr from a partially migratory population with associated scale (100 mm)

The calculated area specific population level reproductive output was higher below than above barrier, suggesting that residents of partially migratory charr populations experience a higher availability of eggs to forage on (Fig. 6, K-W test: X^2^ = 4.333, df = 1, pairwise comparisons using Wilcoxon rank test and bonferroni correction: p-value = 0.037).

#### Stable isotopes

Slope coefficients of linear regression of δ^15^N against standard length were significantly higher in resident charr living below barriers than above barriers (Fig. 2b; Additional file 1: Table S15, LMM: df = 6, *p* = 0.012). Illustrations of the relationship between δ^15^N and standard length for all streams can be found in supplementary material (Additional file 2: Figure S2).

### Back-growth calculation

**Fig. 7:**
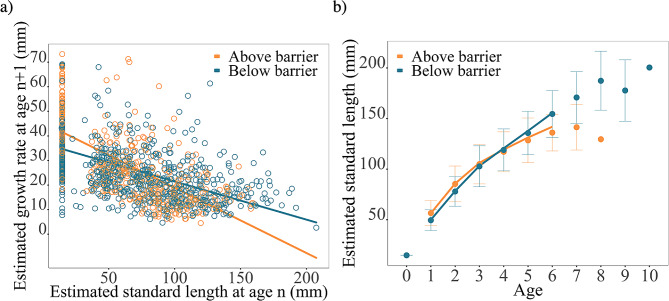
**(a)** Estimated back calculated growth at age *n* + 1 of Arctic charr in relation to their estimated standard length at age n between above and below populations and **(b)** Average estimated standard length at age. Response curves represent predicted values generated from a linear mixed model until age 6

The interaction between barrier and standard length at age n in the linear mixed model indicates that the growth differences between above and below barriers resident charr depend on their standard length, (Fig. 7a; Additional file 1: Table S16, LMM: df = 958.37, *p* < 0.001). Early in life small charr grew faster above compared to below barrier. However, after reaching a certain size, resident charr below barriers grew faster compared to fish above barriers (Fig. 7a). Figures of all streams can be found in supplementary material (Additional file 2: Figure S13).

Resident fish living above barriers are larger in the first and second year of their life (Fig. 7b; Additional file 1: Table S17, LMM: age 1: df = 834.52, *p* < 0.001 & age 2: df = 971.86, *p* < 0.001). In the third and fourth year of life, the two groups do not show differences in estimated standard length (Fig. 7b; Additional file 1: Table S17, LMM: age 3: df = 931.42, *p* = 0.136 & age 4: df = 746.01, *p* = 0.438) but fish living below barriers show larger estimated length in the following years (Fig. 7b; Additional file 1: Table S17, LMM: age 5: df = 634.71, *p* = 0.002 & age 6: df = 652.13, *p* < 0.001). Figures of all streams can be found in supplementary material (Additional file 2: Figure S14).

## Discussion

Our replicated comparative study of riverine food webs separated by migration barriers emphasized the importance of diverse energy and nutrients pathways entering the food web via migration. Our findings highlight how seasonal movements influence the prey consumption and growth of resident individuals. Resident charr (non-migratory individuals from partially migratory populations) cannibalize on eggs and juveniles of migratory individuals. In this way, marine-derived resources enter the local food web at a relatively high trophic level. The availability of these marine-derived resources, ultimately results in relatively large resident individuals that act as a fourth trophic level in the system. We found a larger increase in δ^15^N-values with individual size in ecosystems with migration compared to systems without, which adds to the evidence for a migration-induced elongated food chain. Large resident individuals in partially migratory populations experience reduced intraspecific competition for resources due to their distinct capability to prey on juveniles, thereby potentially balancing their fitness with migrating individuals.

Small resident charr in our studied riverine ecosystems primarily feed on aquatic invertebrates, which are consumed in similar amounts in sections with and without migration. Still, we documented a lower early-life growth of residents exposed to migration, which aligns with our hypothesis. This may be due to higher energy use below the barrier (e.g. associated with more active search for food, aggressive intraspecific interactions or predator avoidance) [63, 64] causing fish to grow slower [65]. As opposed to resident individuals living above barriers, aquatic invertebrate intake of residents living below barriers decreases significantly as they get larger. This suggests that they potentially switch to different available resources. We also acknowledge that other potential environmental variables, which could differ between upstream and downstream stretches may co-affect this pattern. These variables could include lower prey availability downstream and temperature variations although we did not measure those across years but to minimize the effects of biotic and abiotic variables, we sampled upstream and downstream stretches close to the barrier.

Our study shows that rivers lacking marine-derived resources (upstream barriers) harbor resident individuals that feed on terrestrial prey more commonly (e.g. arachnids, worms) compared to residents below barriers with the availability of marine-derived resources. Species exclusively dwelling on land tend to possess lower quantities of specific lipids, such as eicosapentaenoic acid, compared to insect species that undergo an initial aquatic life stage [66]. Consequently, terrestrial prey represent a lower nutritional quality as food for consumers compared to aquatic invertebrates. Low consumption of such prey in partially migratory populations could suggest that their availability differ across upstream and downstream stretches or that they do not represent high growth advantages and are utilized through an opportunistic feeding behavior in rivers where marine-derived resources are not present.

Our study further demonstrates that resident fish in rivers influenced by migration feed on conspecific eggs late in the season. Binge feeding on eggs associated with spawning has been shown in salmon [67] and might have an impact on the growth of individuals as they are known to be of high energy content [29]. Research has shown that salmonid eggs constitute a large proportion of the diet of individual Atlantic salmon (*Salmo salar*) and brown trout (*Salmo trutta*), and that probability of egg foraging increases with the size of the fish, highlighting its importance for adult dietary needs as well [68]. Egg cannibalism was not found in the strictly resident populations, likely since egg density in the absence of anadromous charr is too low for eggs to represent a sustainable food resource as opposed to aquatic- and aerial insects, although it cannot be completely excluded. The high influx of eggs to the resident habitat can thus have opposing effects on residents, depending on their size, by acting as both future competitors and contemporary prey. As we predicted, high densities of juveniles were observed in our study and in other partially migratory populations such as *Oncorhynchus mykiss* [33] and *Salmo trutta* [64], such pattern occurs frequently as migratory females tend to have a high fecundity [69]. Our results demonstrate a higher probability of residents cannibalizing on juveniles when migration occurs in the system confirming our expectations. Arctic charr are recognized for their cannibalistic tendencies in lakes [70–73]. Such behavior is also found in rivers [74], which is also the case in our study, when fish reach a suitable size and encounter a high abundance of smaller conspecifics. Migratory animals can alter local communities and ecosystem dynamics through indirect effects by serving as carriers for diseases, nutrients, and energy across habitats [75]. Migrating fish transfer nutrients such as nitrogen and phosphorus to freshwater environments during spawning and subsequent decomposition [76–78], these substances function as fertilizers for primary producers impacting the abundance and diversity of organisms throughout the food web [78–80]. Our findings show that energy can also take alternative pathways and be transferred through the direct consumption of salmonid eggs and juveniles that are available for organisms at higher trophic levels.

The majority of fish species undergo changes in their diets as they mature, transitioning from consuming small prey to larger ones as they grow. Therefore, the presence of appropriate prey during early stages of development is critical for this transition. Our findings indicate that charr consume migration-associated resources such as eggs and newly hatched larvae and that they start to benefit from these resources relatively early in life. In addition to having suitable food resources, the shift towards consuming larger diet items requires the predator to reach a size sufficient to effectively consume fish as prey. Our study shows that once charr are able to cross a certain size-related threshold in relation to their prey, they start consuming larger juveniles, which are available year-round, as opposed to eggs and larvae only present in the time of spawning and emergence. Ontogenetic niche shifts are defined as changes in habitat or resource use as an organism grows in size [81]. These shifts often align with distinct periods of growth that occur at crucial points in the life history of organisms [82, 83]. Growth rate can be influenced by both the size of predators and prey. For instance, in the case of largemouth bass (*Micropterus salmoides*), smaller individuals displayed slower growth rate when feeding on smaller prey, while their growth increased with size as they progressively consumed larger prey [84]. Our study further shows that residents have a higher growth rate in the presence of migratory individuals only after reaching a certain size compared to residents from above barriers. Such pattern suggests that non-migratory individuals from partially migratory populations benefit greatly from egg and fish larvae consumption at small size giving an initial growth increase, which then enables the large residents to start feeding on larger prey enhancing their growth furthermore. Increase in growth rate has been shown for example in resident trout when they consume resources originating from salmon such as eggs [67].

We show that migrants have both negative and positive effects on the non-migratory individuals, which may explain why partial migration is such a common strategy among Arctic charr. We observed that early in life, growth rates are negatively affected in the presence of migrants, possibly due to the high density of juveniles produced by returning females. Such a decrease in growth rate in the presence of migrants, may negatively affect the fitness of non-migratory individuals. This can result in selection of a higher migration propensity and ultimately full migration. However, later in life, egg cannibalism may compensate for the negative effects of competition, but if a large part of the population forages on this very seasonal resource, it is unlikely that it would halt the positive feedback loop promoting migration. Migration provides greater feeding opportunities but it also entails high predation risk [85]. Therefore, a significant decrease in competition in the habitat of origin should promote residency. The exclusive ability of larger residents to prey on juveniles, which are abundant, grants them access to a resource without competing with the offspring of potentially future migratory individuals. This interruption in the positive feedback loop may likely level the fitness of residents and migrants. Such forms of resource partitioning that prevent competition between migrants and non-migrants can thus lead to the establishment of partial migration as an evolutionary stable strategy [86–88]. Our findings suggest that further research could delve into examining the fitness disparities between strategies in partially migratory populations. One potential approach could involve investigating the long-term survival rates and reproductive success of large residents and migrants across replicated streams. If different life-history strategies can produce similar lifetime fitness values in Arctic charr populations, it raises the question why there are still many species of salmonids (e.g. salmon species) fully migratory. In our study system only one fish species is present, therefore there is no interspecific competition, which is rarely the case in other systems. In habitats occupied by multiple species, dietary niches might be already filled, thereby limiting the possibilities of new feeding opportunities for resident individuals. For example, in Pacific salmon (*Oncorhynchus*), rainbow trout is feeding on *Oncorhynchus nerka* eggs [67], while Bull trout is foraging on juvenile *Oncorhynchus tshawytscha* [89].

Hence, juvenile fish represent an additional energy pathway to the food web in the presence of migration. The emergence of larger resident charr in partially migratory populations feeding on smaller ones suggest that an additional trophic level might be present. This hypothesis is not only supported by larger sizes of charr and the commonness of cannibalism, but also by an increase in δ^15^N in the fish tissues as individuals get larger. δ^15^N tends to increase in the tissues of organisms as they move up the trophic levels of a food chain [90]. Therefore, the relationship of δ^15^N with individual size can reflect changes in the trophic structure of the community. Our study shows that steeper δ^15^N-size slopes are present in systems influenced by marine-derived resources, indicating changes in dietary preferences. A steeper slope suggests a more pronounced increase in trophic position δ^15^N with size. Such findings are supported by results from another study focusing on only one of the streams in our system, where amino acid δ^15^N shows that increase of bulk δ^15^N is reflective of an increase of trophic position [91].

Those findings support the hypothesis that direct consumption of eggs and juveniles favor the presence of successively larger predators, and consequently, longer food chains. While our study provides strong evidence supporting the inclusion of an additional trophic level, we could not estimate trophic position due to lack of isotopic baselines. Maximum trophic position could have allowed us to quantify and compare food chain length between above and below populations, therefore studies with detailed baseline corrections could be useful to potentially support our hypothesis. Although our study design allows for replications between streams, some variability in the magnitude of the effects have to be taken into consideration. The abiotic conditions of the streams (e.g. temperature, substrate composition, flow velocity) and seasonal fluctuations of prey can affect growth and survival of young fish [92, 93], which might prevent the processes leading to elongation of food chain length to happen in all streams. Therefore, research on such factors would be beneficial to understand how those processes can be dampened.

Literature often focuses on the impact of energy and nutrient transfer from anadromous fish on ecosystems through bottom-up pathways [94], potentially diverting attention from other crucial ecosystem functions they may serve. Our work demonstrates how a migratory predator species is able to create a middle-up food web effect by adding resources (e.g. eggs, juveniles) in environments that can be consumed by resident conspecifics at early and late life stages. Direct access to such resources leads potentially to an elongated food chain length possibly balancing the fitness of large resident individuals with migratory ones. Such phenomena raise the question about the importance of energy pathways in the evolution and maintenance of partial migration. In our study, the ecological consequences of seasonal movements of migratory individuals are strongly demonstrated in food webs with a single top predator species. To understand the extent of the effect of migration on food web structure, further research should explore ecosystems of greater complexity, where multiple species interact. Our study demonstrates that migration has the capacity to alter food webs through direct pathways, thereby playing a crucial role in shaping ecosystems. Consequently, it is imperative to consider migration when looking at ecological dynamics and the ecology of non-migratory species.

### Electronic supplementary material

Below is the link to the electronic supplementary material.

Supplementary Material 1

Supplementary Material 2

## Data Availability

The datasets generated and/or analyzed during the current study are available in the OSF repository, https://osf.io/92hsg/?view_only=945a7752866448549fb1e0ef0d04b826.
