# Supplementary material for: Migratory-derived resources induce elongated food chains through middle-up food web effects
Source: Mov Ecol. 2024 Aug 20;12:56. doi: 10.1186/s40462-024-00496-4 (PMC11337878; doi:10.1186/s40462-024-00496-4)
Supplement: Supplementary file 1 — Supplementary Material 1 [file 40462_2024_496_MOESM1_ESM.docx]

Authors: Coralie Moccetti, Nicola Sperlich, Grégoire Saboret, Hanna ten Brink, Jakob Brodersen

**Manuscript title:** Migratory-derived resources induce elongated food chains through middle-up food web effects

**Supplementary tables**

**Table S1.** Given names and characteristics of each stream sampled in southern Greenland.

|  | **Altitude range a.s.l (m)** | | **Width (average in m)** | | **Glacial influence** | | **Lake upstream** | |
| --- | --- | --- | --- | --- | --- | --- | --- | --- |
|  | Above | Below | Above | Below | Above | Below | Above | Below |
| Qinnguata Kuua | 210 | 65 | Unknown | Unknown | Yes | Yes | No | No |
| Ermata Kuua | 107 | 73 | 4.7 | 6.9 | No | No | Yes | Yes |
| Qorlortoq Kuua | 157 | 34 | 8.8 | 9.4 | No | No | No | No |
| Qassiarsuk Kuua | 74 | 36 | 2.1 | 1.9 | No | No | Yes | Yes |
| Qorlortup Itinnera Kuua | 74 | 32 | 6.9 | 6.7 | No | No | No | No |
| Tasiusaq Kuua | 166 | 57 | 1.6 | 1.7 | No | No | Yes | Yes |
| Nunataaq Kuua | 69 | 54 | 1.1 | 3.5 | No | No | Yes | Yes |

Abbreviations: m: meters, a.s.l.: above sea level

**Table S2.** Composition of prey found in the stomachs of resident charr.


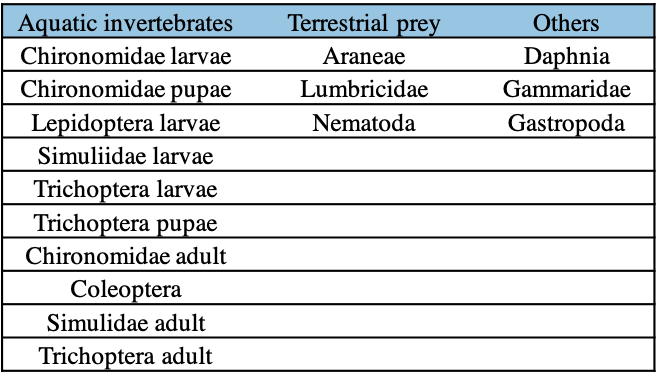


**Table S3.** Number of resident charr otoliths analyzed for back-growth calculation per stream above and below barriers.

|  | Qinnguata Kuua | Qassiarsuk Kuua | Ermata Kuua | Qorlortoq Kuua | Qorlortup Itinnera Kuua | Tasiusaq Kuua | Nunataaq Kuua |
| --- | --- | --- | --- | --- | --- | --- | --- |
| **Above** | **6** | **19** | **22** | **23** | **25** | **10** | **23** |
| **Below** | **23** | **28** | **8** | **28** | **20** | **20** | **22** |

**Table S4.** Poisson-distributed Generalized Linear Mixed Effects Models (GLMM) used to analyze the aquatic invertebrate abundance estimated in 2021 from each stream above and below barriers. The model evaluates the impact of the relative position to the barrier (upstream vs. downstream) on aquatic invertebrate abundance while accounting for the variability among streams. We show the incidence rate ratios (IRR) and confidence intervals (CI), as well as the p-values (p). An IRR greater than 1 indicates a higher event rate compared to the reference group.

**
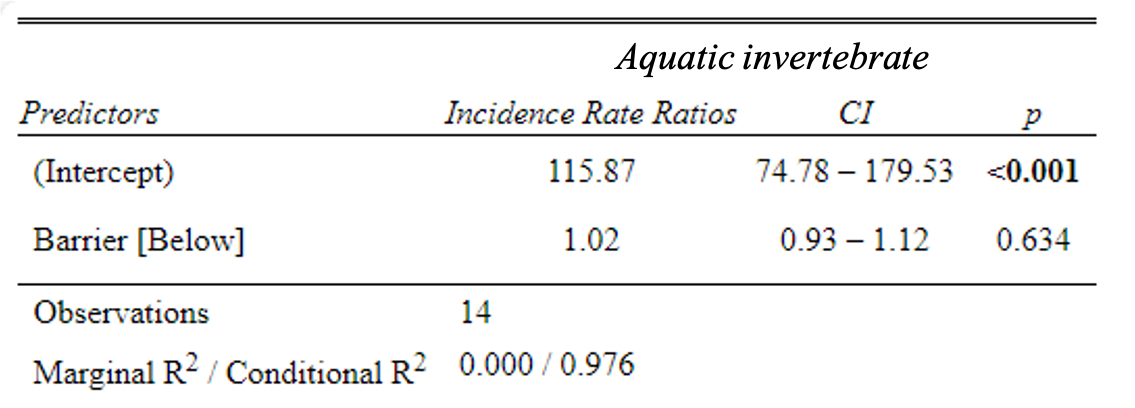
**

**Table S5.** Coefficient estimates fitting a linear mixed model applied to stream width estimated in 2021 from each stream above and below barriers. Stream was assigned as a random factor. We show the estimates and confidence intervals (CI), as well as the p-values (p) and degrees of freedom (df). P-values were estimated with the Kenward-Roger approximation to adjust the degrees of freedom.

**
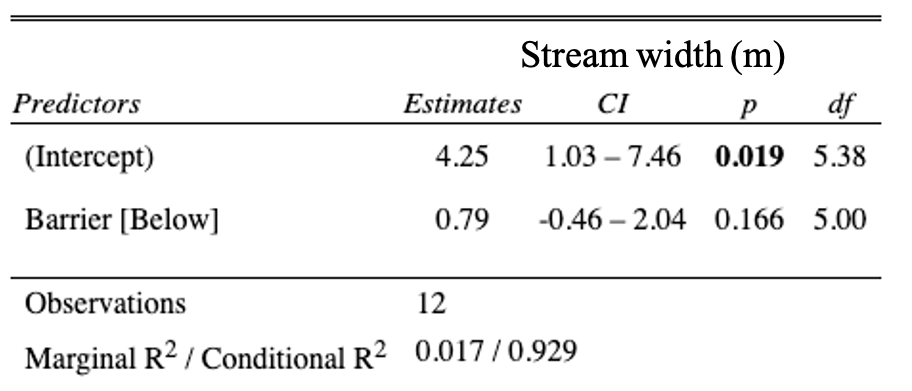
**

**Table S6.** Poisson-distributed Generalized Linear Mixed Effects Models (GLMM) used to analyze the density of juvenile charr (individuals/ m^2^) from each stream above and below barriers (nAbove= 38, nBelow= 163). The model evaluates the impact of the relative position to the barrier (upstream vs. downstream), aquatic invertebrate abundance and stream width on fish density while accounting for the variability among streams and adjusting for the area of the stream sections. We show the incidence rate ratios (IRR) and confidence intervals (CI), as well as the p- values (p). An IRR greater than 1 indicates a higher event rate compared to the reference group.

**
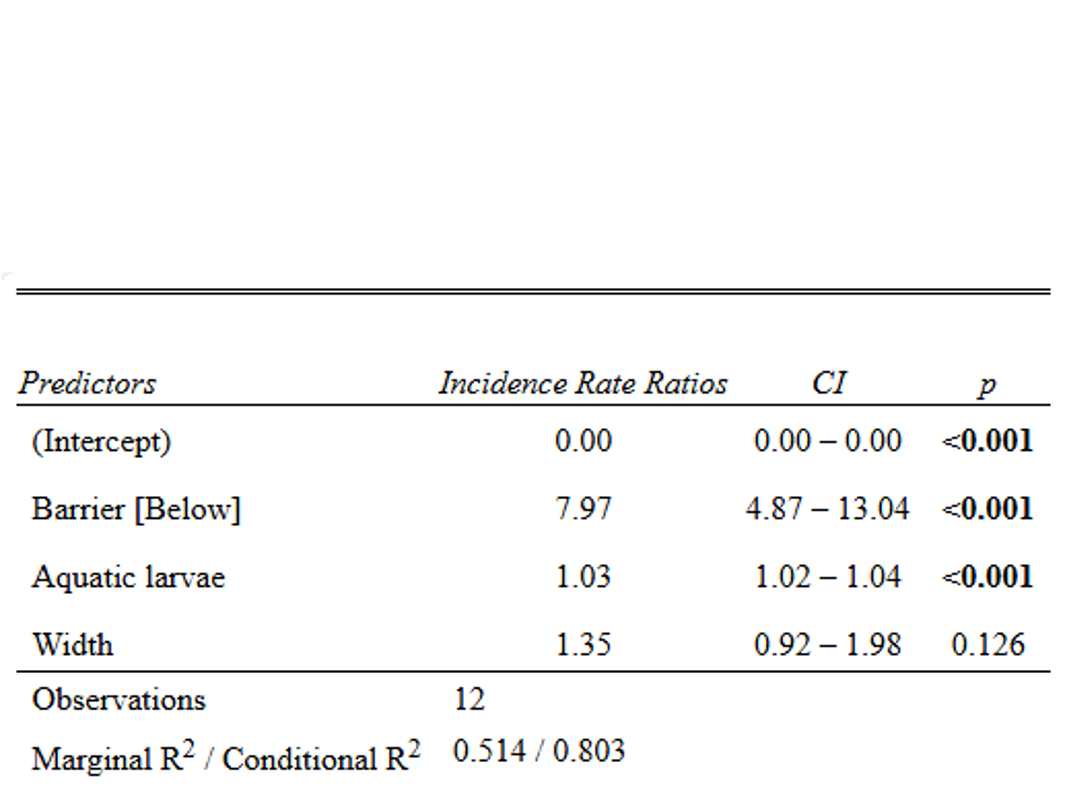

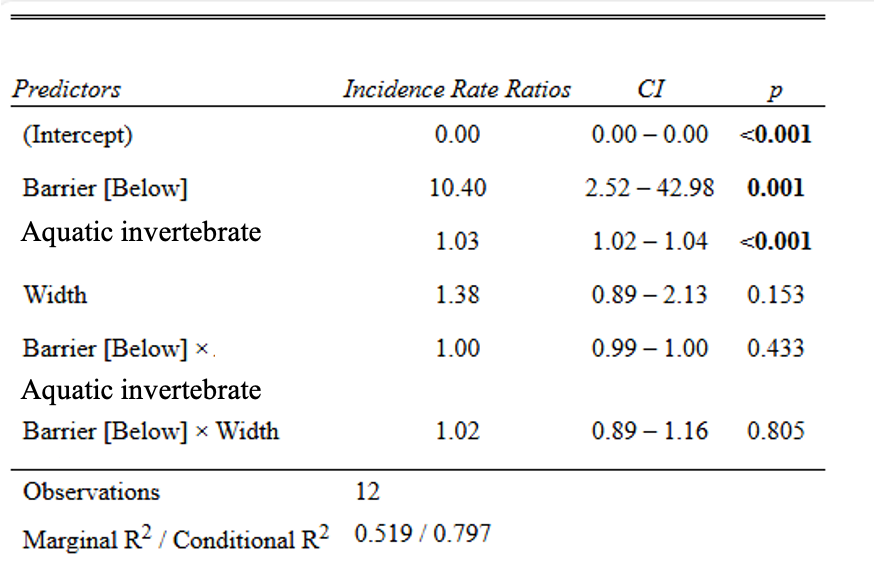
**

**Table S7.** Poisson-distributed Generalized Linear Mixed Effects Models (GLMM) used to analyze the density of resident male charr (individuals/ m^2^) from each stream above and below barriers (nAbove= 38, nBelow= 163). The model evaluates the impact of the relative position to the barrier (upstream vs. downstream), aquatic invertebrate abundance and stream width on fish density while accounting for the variability among streams and adjusting for the area of the stream sections. We show the incidence rate ratios (IRR) and confidence intervals (CI), as well as the p-values (p). An IRR greater than 1 indicates a higher event rate compared to the reference group.


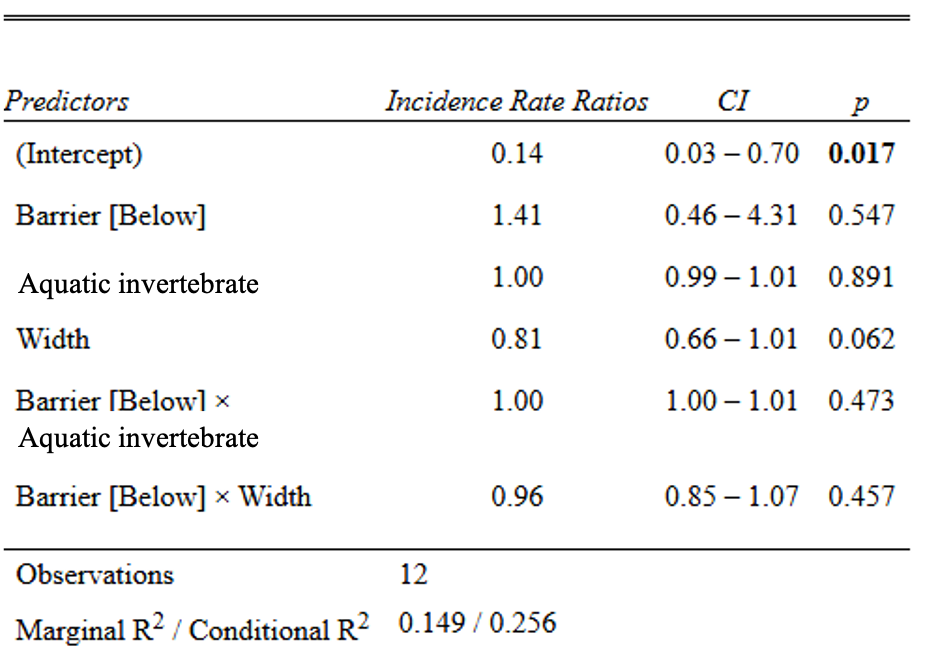


**Table S8.** Poisson-distributed Generalized Linear Mixed Effects Models (GLMM) used to analyze the density of resident female charr (individuals/ m^2^) from each stream above and below barriers (nAbove= 38, nBelow= 163). The model evaluates the impact of the relative position to the barrier (upstream vs. downstream) on fish density while accounting for the variability among streams and adjusting for the area of the stream sections. We show the incidence rate ratios (IRR) and confidence intervals (CI), as well as the p-values (p). An IRR greater than 1 indicates a higher event rate compared to the reference group.


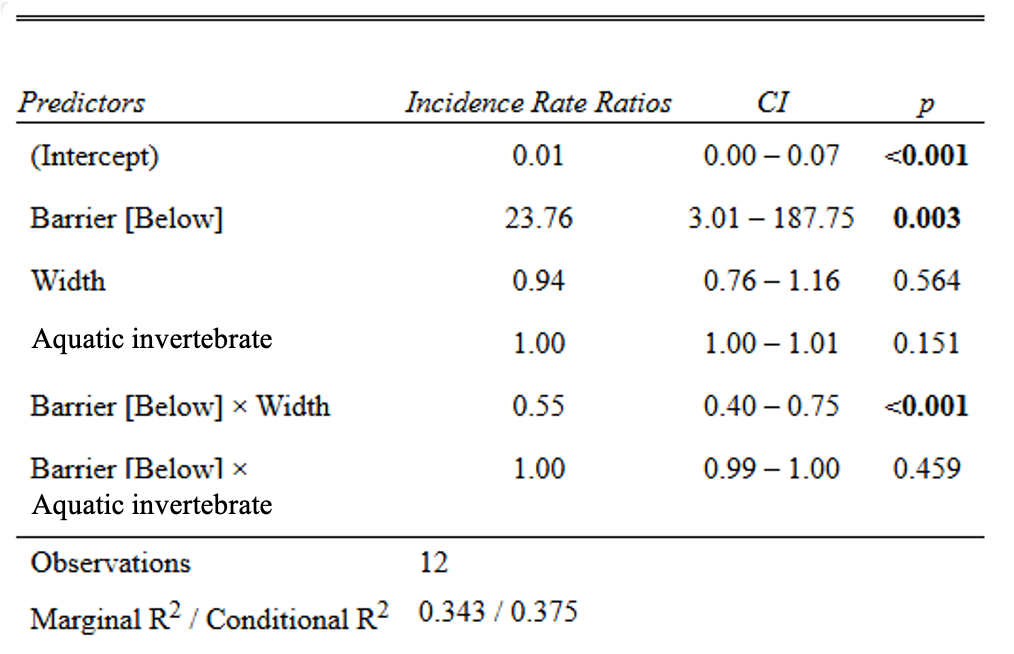


**Table S9.** Coefficients from a quantile regression model that estimates the 80^th^ percentile of charr (juveniles and residents) standard length as a function of barrier.

| Coefficients | Value | Standard error | t value | P value |
| --- | --- | --- | --- | --- |
| Intercept | 144 | 2.200 | 65.434 | <0.001 |
| Barrier (below) | 15 | 3.914 | 3.831 | <0.001 |

**Table S10**. Mixed effects logistic regression model applied to proportions of resident females from above and below barriers. Stream was assigned as a random factor. We show the odds ratios (OR), confidence intervals (CI), as well as the p-values (p). The estimate indicates the direction of effect (increase or decrease in log-odds), while the odds ratios quantify the magnitude of this effect. A negative estimate and corresponding odds ratio less than 1 suggest that the location of the barrier is associated with a lower likelihood of observing females below the barrier.

**
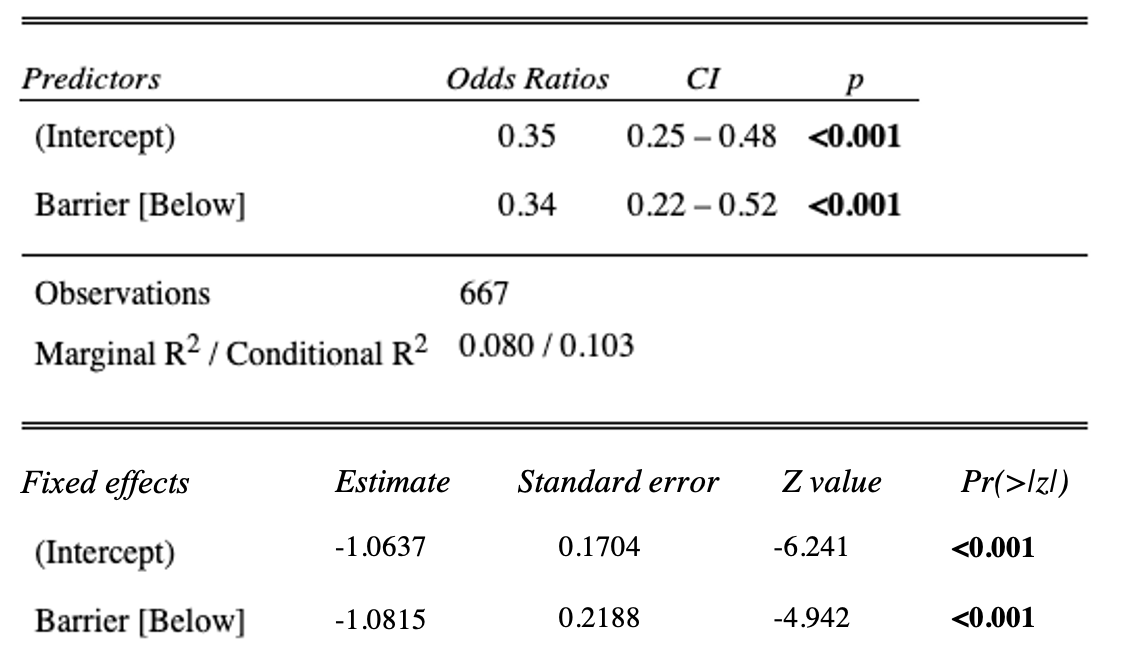
**

**Table S11.** Pairwise differentiation table showing values of Fst for all pairs populations living above and below the barriers. All comparisons were significants (p<0.001). ER1: Qinnguata Kuua, ER3: Ermata Kuua, ER4: Qorlortoq Kuua, ER10: Qassiarsuk Kuua, SR1: Qorlortup Itinnera Kuua, SR2: Tasiusaq Kuua, SR4: Nunataaq Kuua.


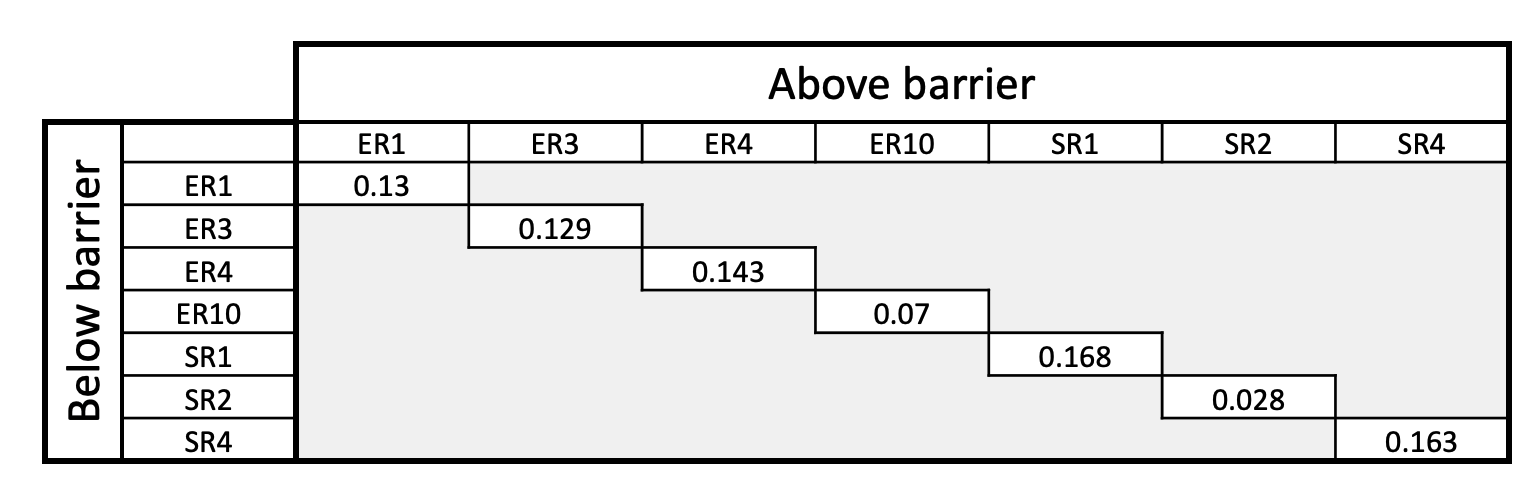


**Table S12.** Likelihood ratio test performed on a logistic regression model to test the occurrence of resident charr feeding on terrestrial prey between populations living above and below barriers.


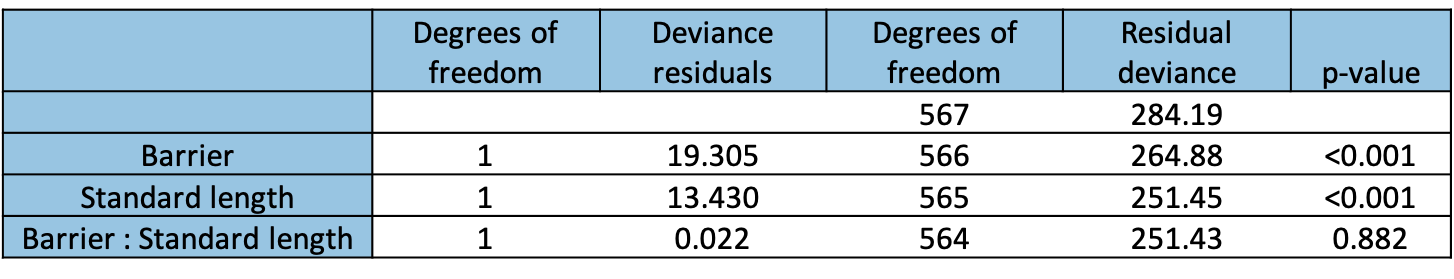


**Table S13.** Likelihood ratio test performed on a logistic regression model to test the occurrence of resident charr feeding on charr eggs between populations living above and below barriers.


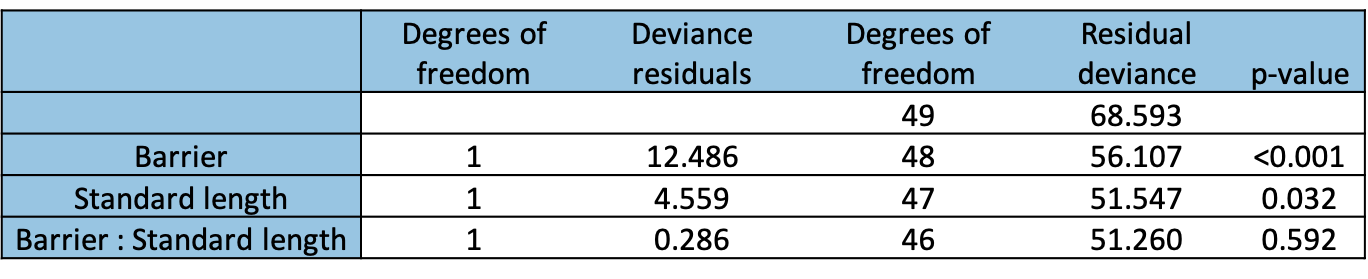


**Table S14.** Likelihood ratio test performed on a logistic regression model to test the occurrence of resident charr feeding on juveniles between populations living above and below barriers.


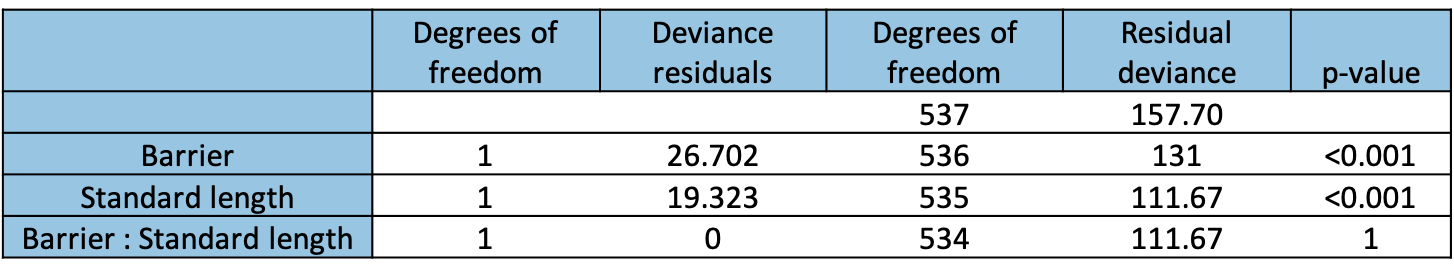


**Table S15.** Coefficient estimates fitting a linear mixed model applied to slope coefficients of linear regression of δ^15^N against standard length from each stream above and below barriers (nAbove= 24, nBelow= 21). Stream was assigned as a random factor. We show the estimates and confidence intervals (CI), as well as the p- values (p) and degrees of freedom (df). P-values were estimated with the Kenward-Roger approximation to adjust the degrees of freedom.


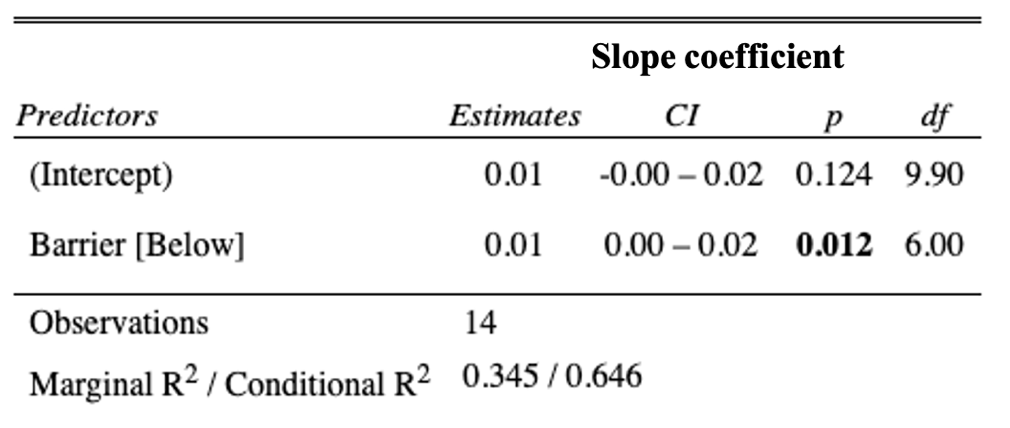


**Table S16.** Coefficient estimates fitting a linear mixed model applied to estimated growth of resident charr of different sizes (Est_length) from above and below barriers. Stream and individual were assigned as random factors. We show the estimates and confidence intervals (CI), as well as the p-values (p) and degrees of freedom (df). P-values were estimated with the Kenward-Roger approximation to adjust the degrees of freedom.


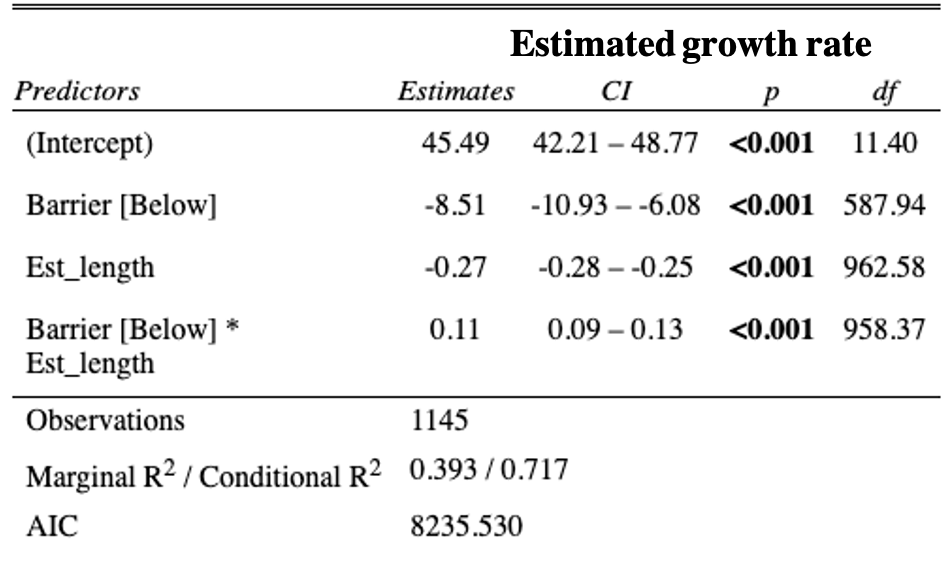


**Table S17.** Coefficient estimates fitting a random slope model applied to estimated length (Est length) of resident charr of different ages (Agei_factor) from above and below barriers. Stream and Individual were assigned as random factors. We show the estimates and confidence intervals (CI), as well as the p-values (p) and degrees of freedom (df). P-values were estimated with the Kenward-Roger approximation to adjust the degrees of freedom.

**
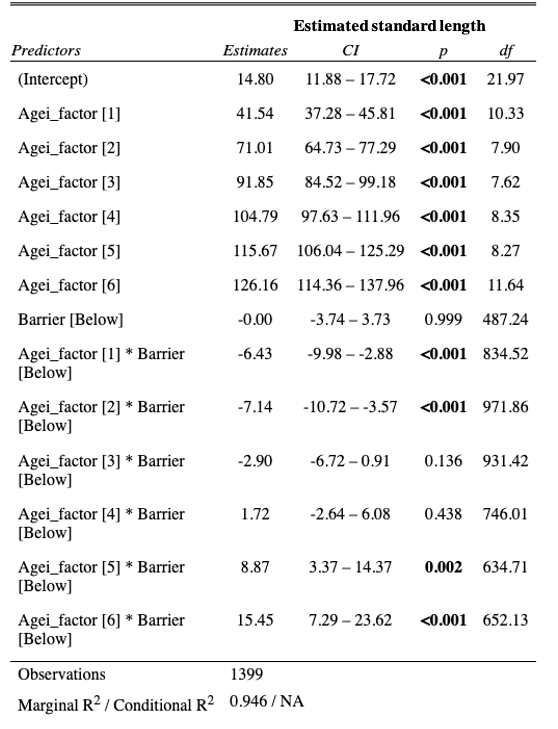
**
