# Supplementary material for: Migratory-derived resources induce elongated food chains through middle-up food web effects
Source: Mov Ecol. 2024 Aug 20;12:56. doi: 10.1186/s40462-024-00496-4 (PMC11337878; doi:10.1186/s40462-024-00496-4)
Supplement: Supplementary file 2 — Supplementary Material 2 [file 40462_2024_496_MOESM2_ESM.docx]

Authors: Coralie Moccetti, Nicola Sperlich, Grégoire Saboret, Hanna ten Brink, Jakob Brodersen

**Manuscript title:** Migratory-derived resources induce elongated food chains through middle-up food web effects

**Supplementary figures**


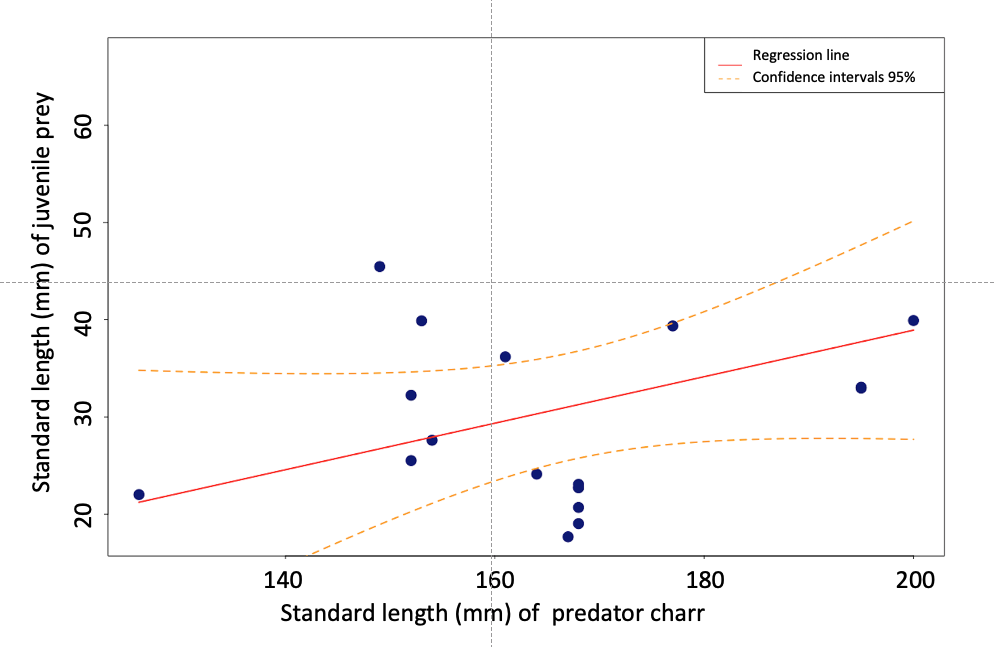


**Figure S1.** Relationship between the standard length of juvenile charr found in the stomach of residents and the standard length of the consumer resident charr.

**
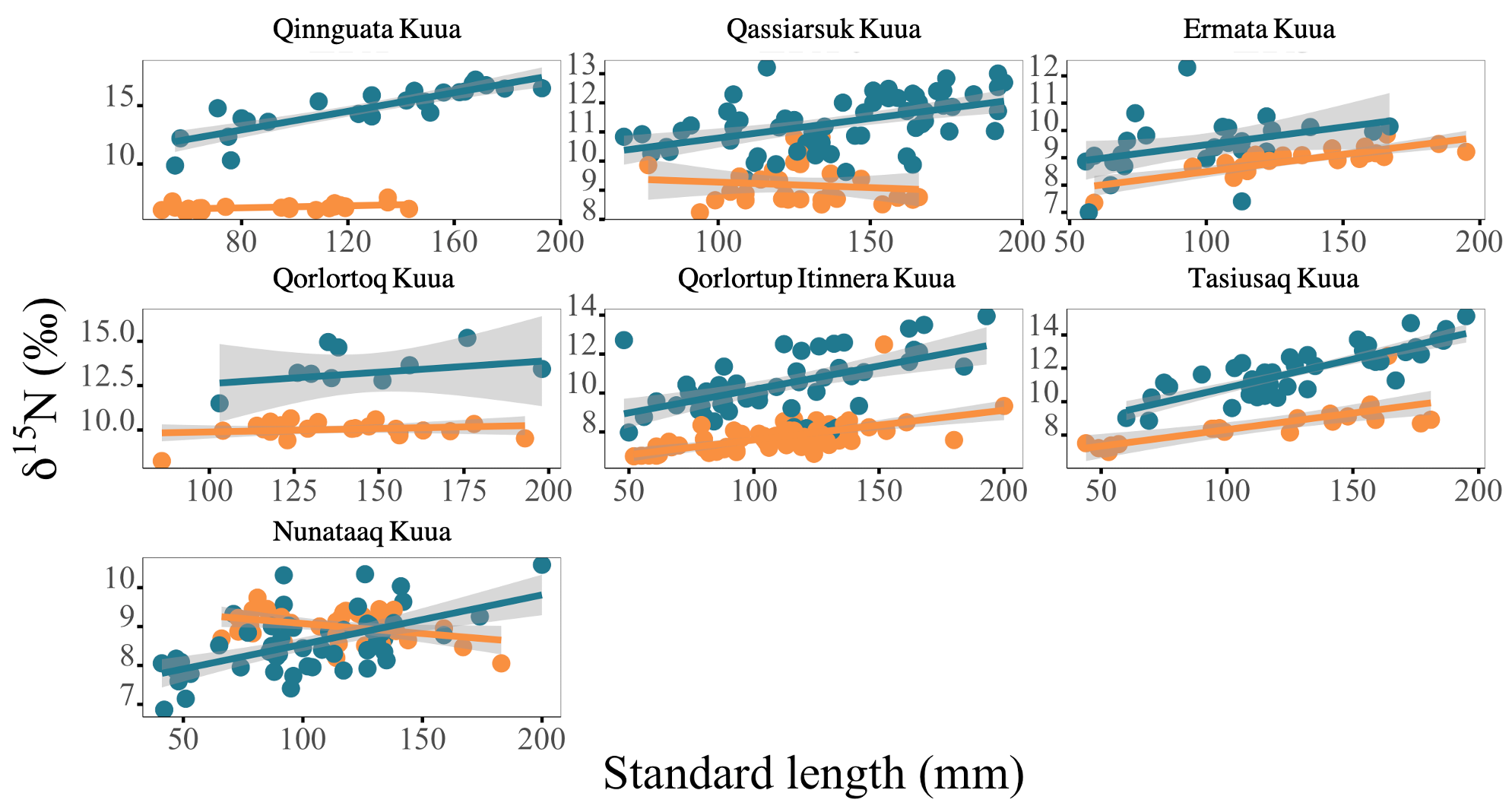
 Figure S2.** Relationship between bulk stable isotopes of nitrogen (y-axis) and standard length of resident Arctic charr (x-axis) per stream between above and below populations. Response curves represent predicted values generated from a linear mixed model.


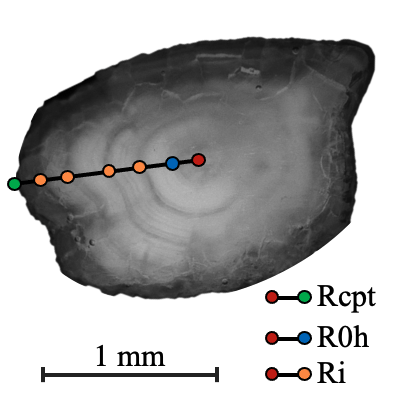


**Figure S3.** Picture of a polished otolith taken under stereo-microscope; each orange circle represents an annuli. The red circle represents the nucleus, the blue circle represents the radius at hatch and the green circle represents the radius at capture. R*_i_* is the otolith radius at age *i* (mm), Rcpt is the otolith radius at capture (mm), R0h the otolith radius at hatch (mm).


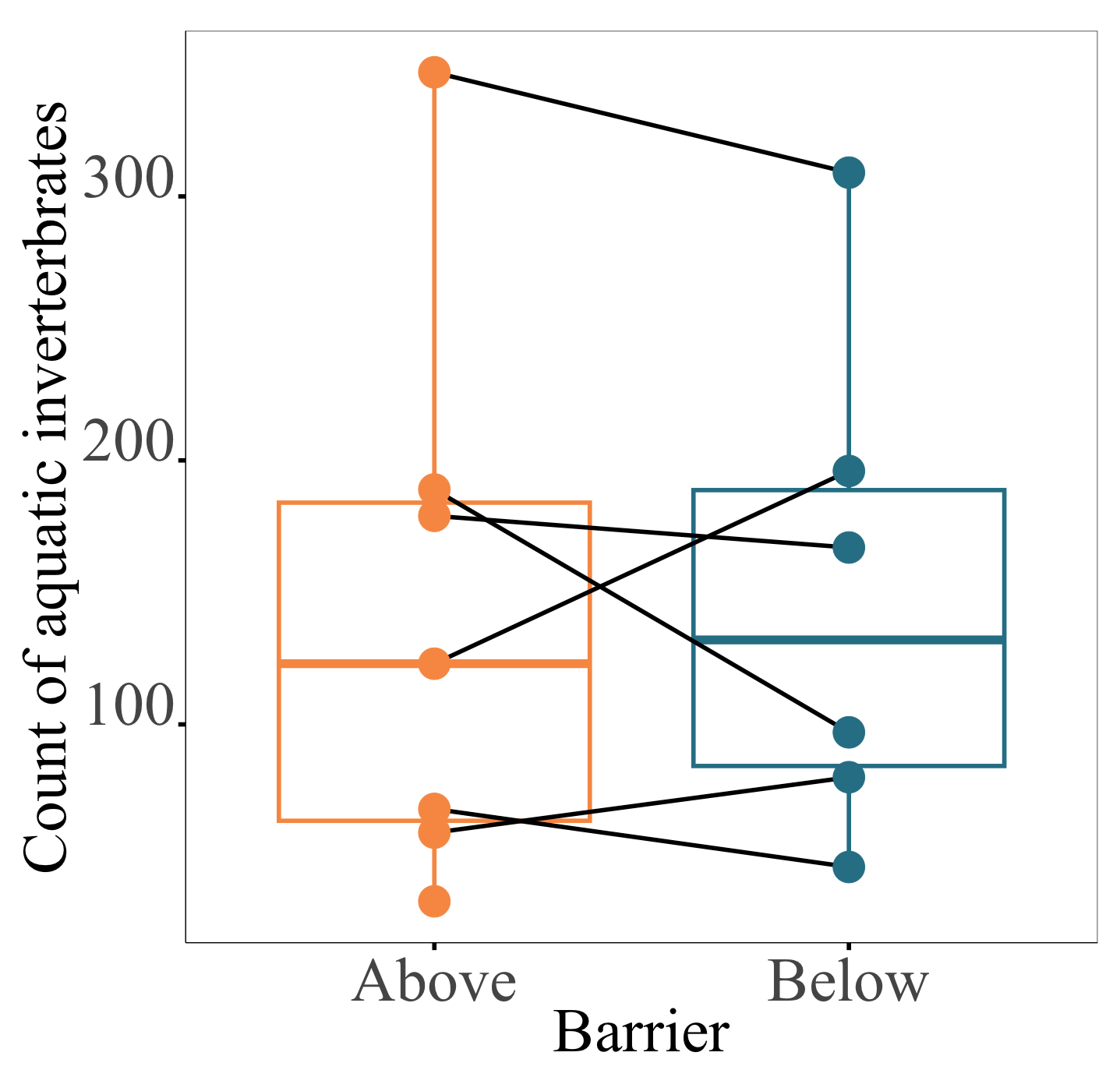


**Figure S4.** Average count of aquatic invertebrates sampled by kick sampling above and below stretches.


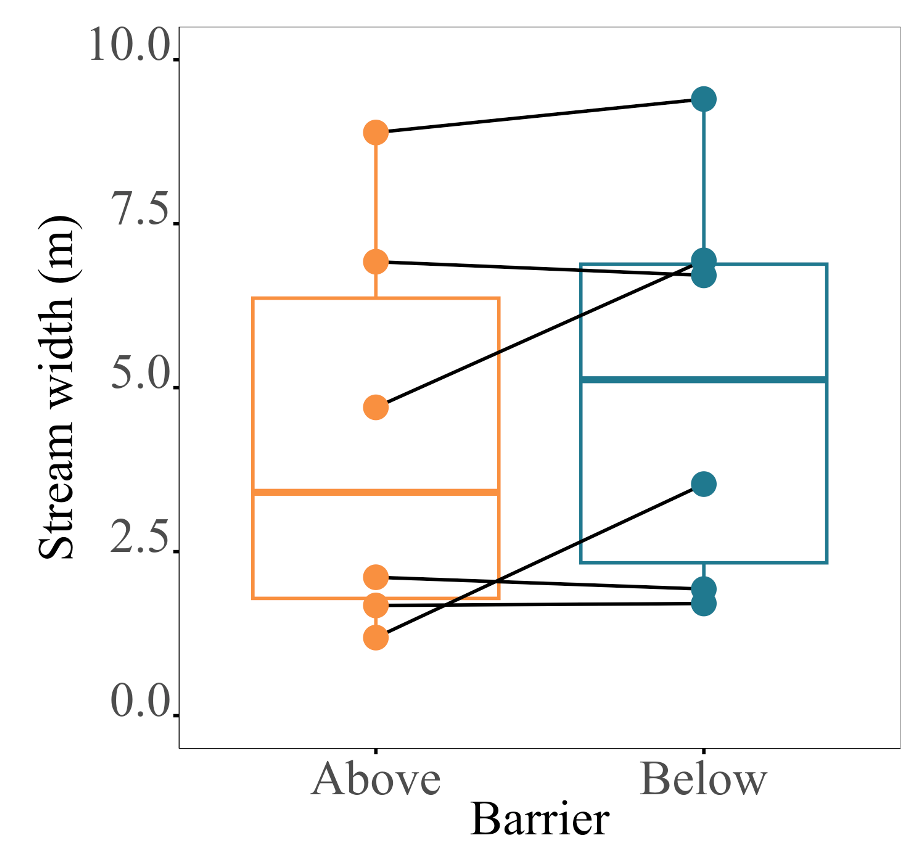


**Figure S5.** Average stream width (m) of above and below barrier stretches sampled.

**
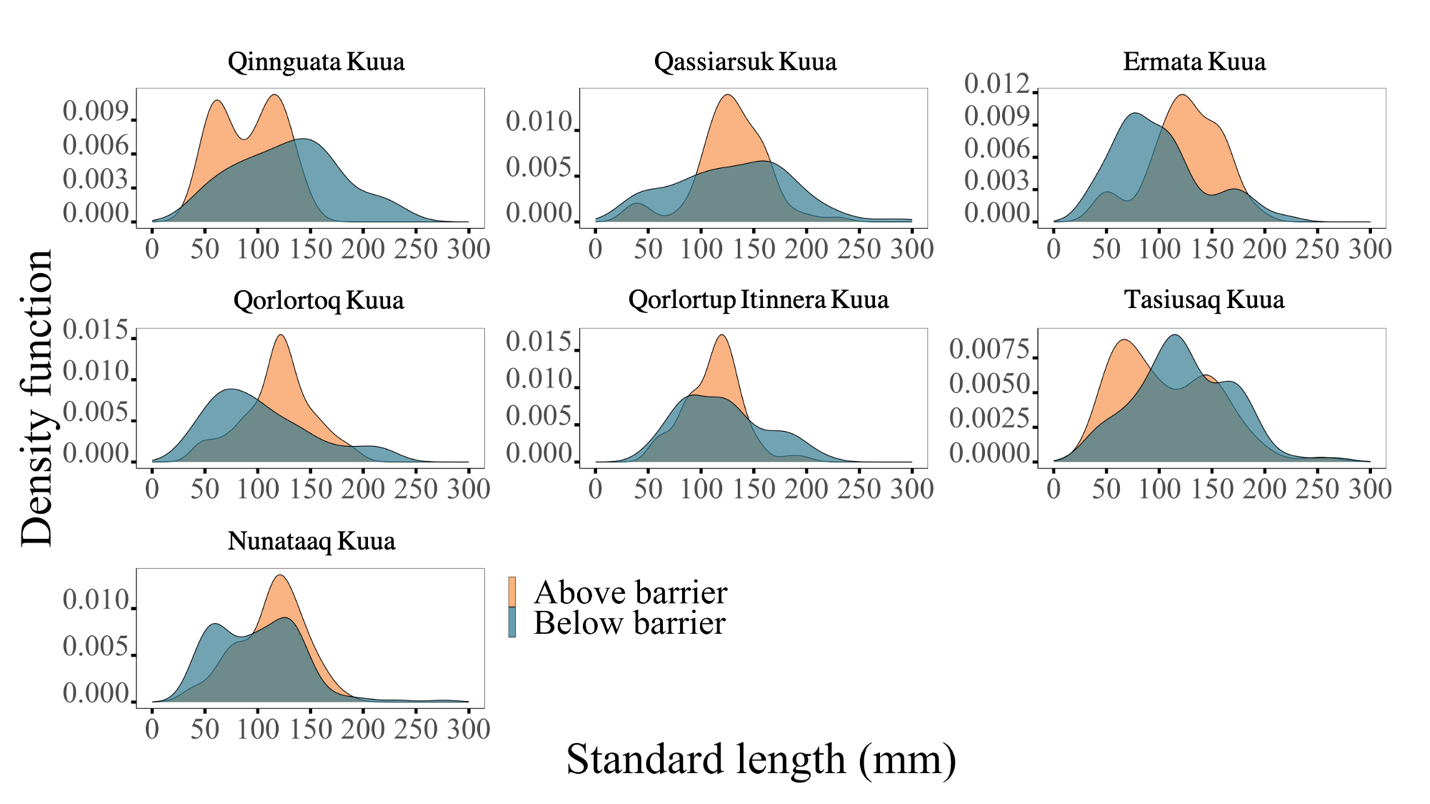
Figure S6.** Comparisons of populations living above and below barriers per stream with Kernel density plots of all residents caught in 2018, 2019 and 2021.


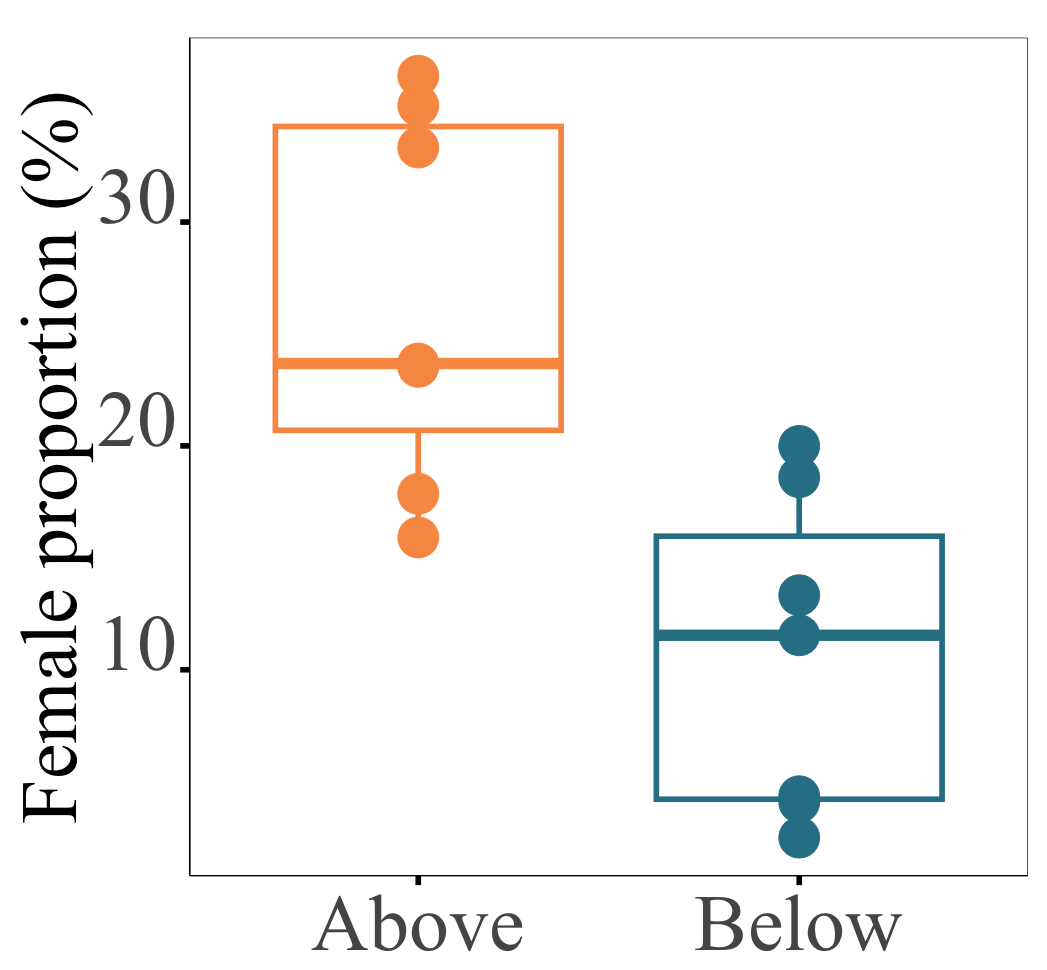


**Figure S7.** Comparison of proportions of resident females sampled above and below barriers in seven streams.


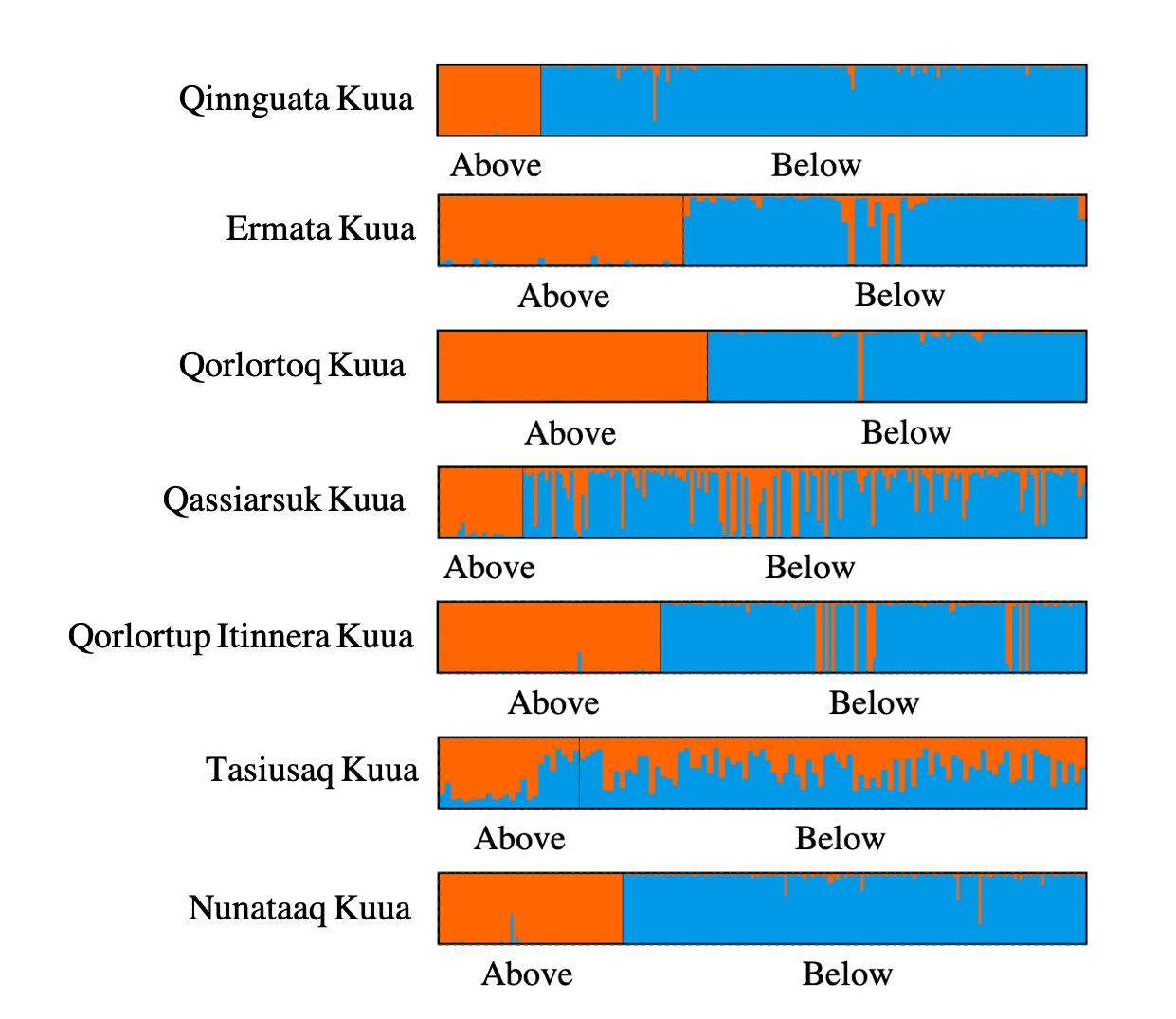


**Figure S8.** Plots of genetic assignment of all streams as obtained in the hierarchical STRUCTURE analysis. We ran above and below barriers populations together with K=1-4, with 10 replicates, 50000 burn-in and Markov Chain Monte Carlo algorithm steps each, applying the admixture model for correlated allele frequencies. Each bar represents the probability of an individual to belong to a genetic cluster.

**
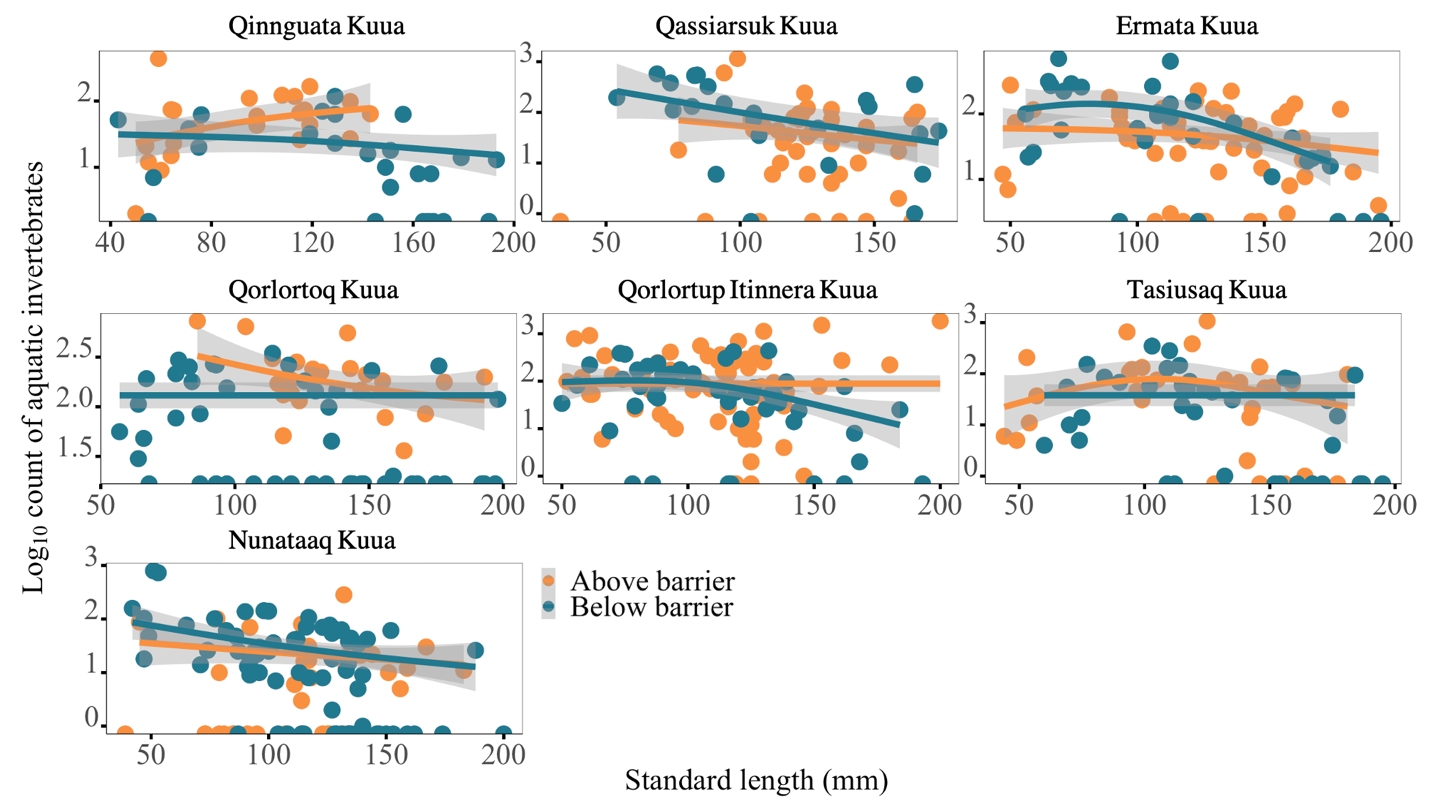
Figure S9.** Log_10_ count of aquatic invertebrates found in the stomachs of Arctic charr in relation to their size for each stream. Response curves represent predicted log_10_ count generated from a Generalized Additive Model (GAM).


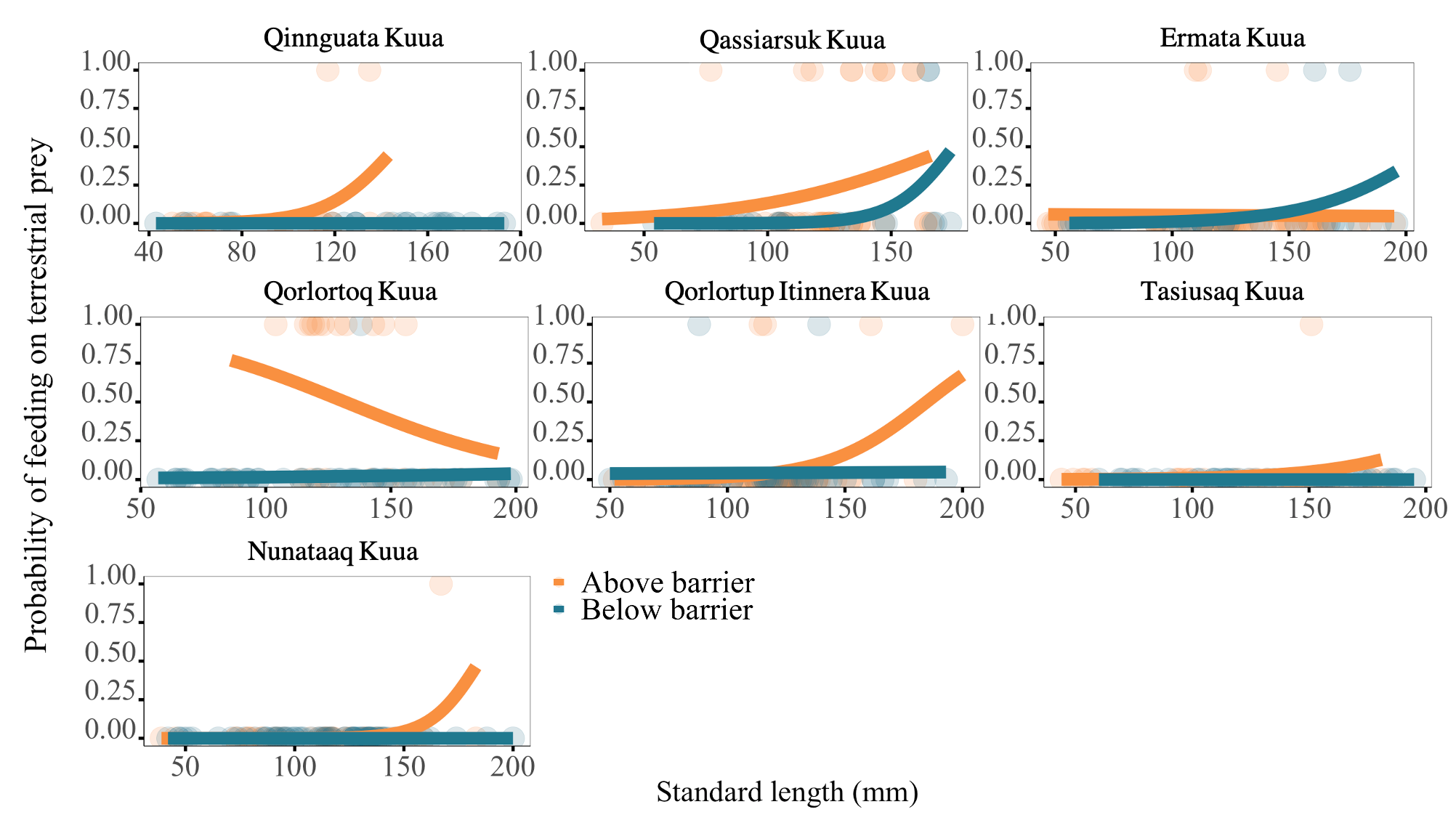


**Figure S10.** Logistic regression (GLM) per stream showing the probability of charr to feed on terrestrial prey according to standard length between populations of above and below waterfalls.

**
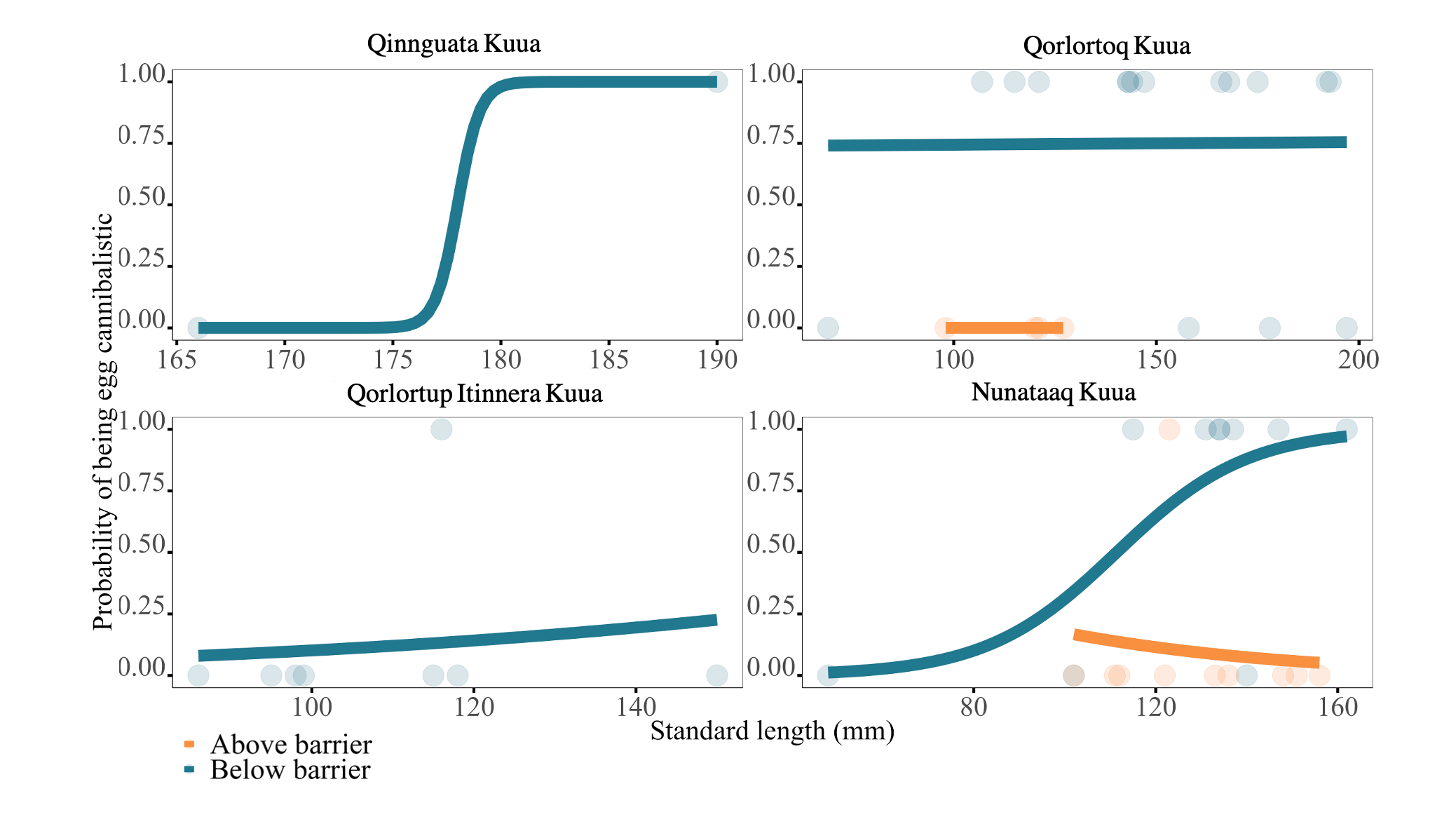
 Figure S11.** Logistic regression (GLM) per stream showing the probability of charr to feed on charr egg (Year 2021) according to standard length between populations of above and below waterfalls.

**
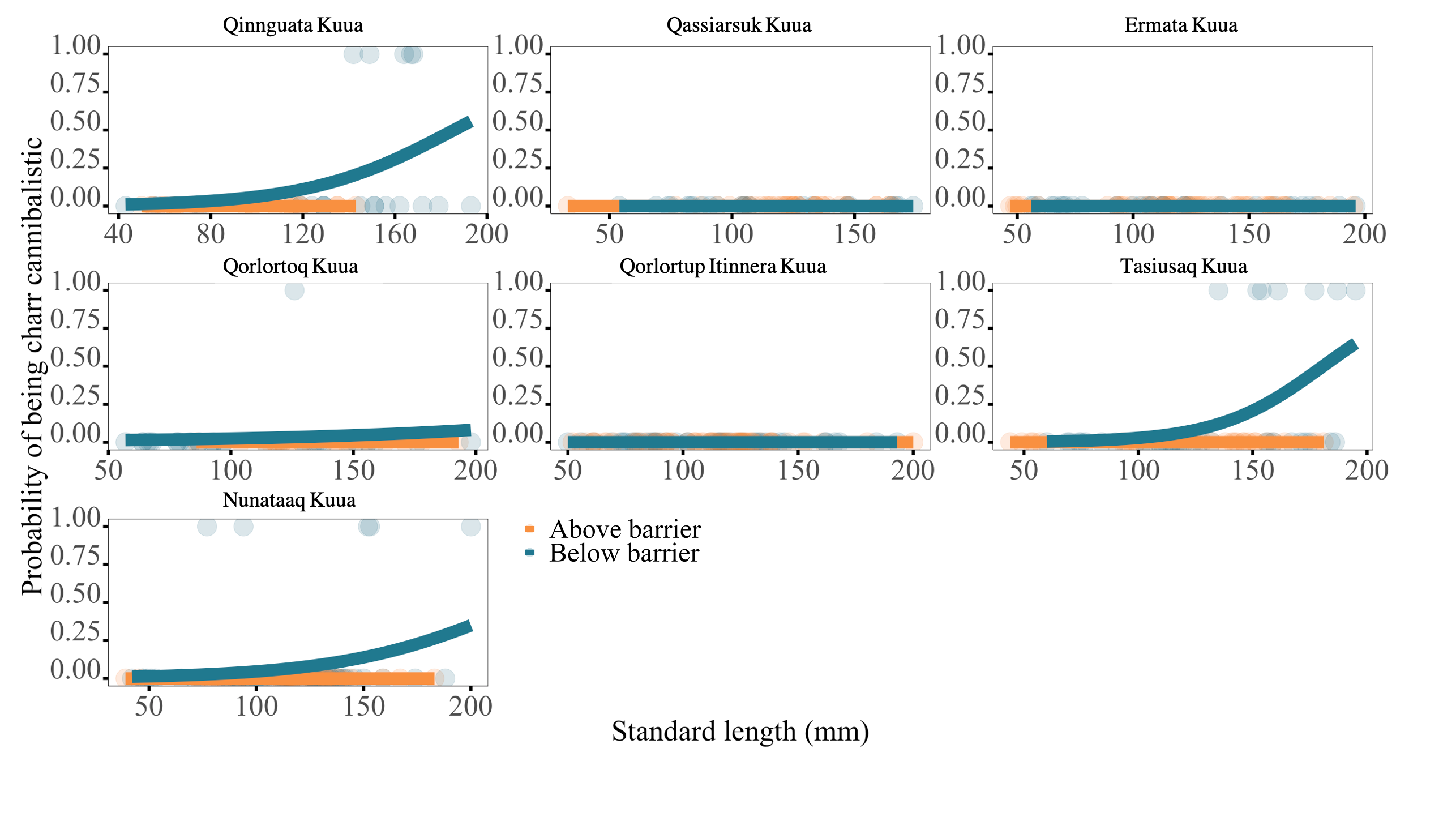
 Figure S12.** Logistic regression (GLM) per stream showing the probability of charr to feed on juvenile charr (Year 2018 and 2019) according to standard length between populations of above and below waterfalls.

**
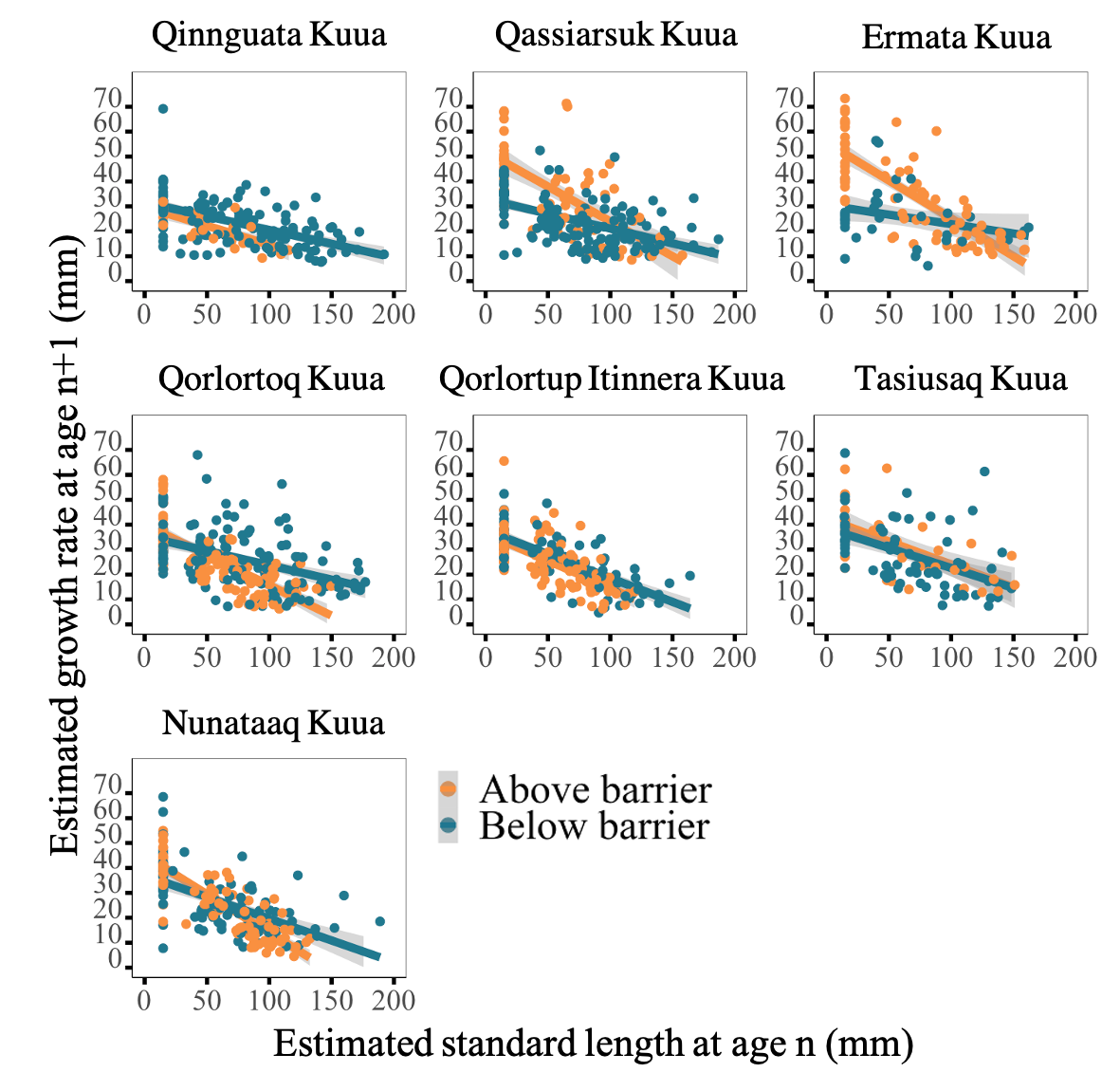
 Figure S13.** Estimated back calculated growth at age n + 1 of Arctic charr per stream in relation to their estimated standard length at age n between above and below barrier populations. Response curves represent predicted values generated from a linear mixed model.

**
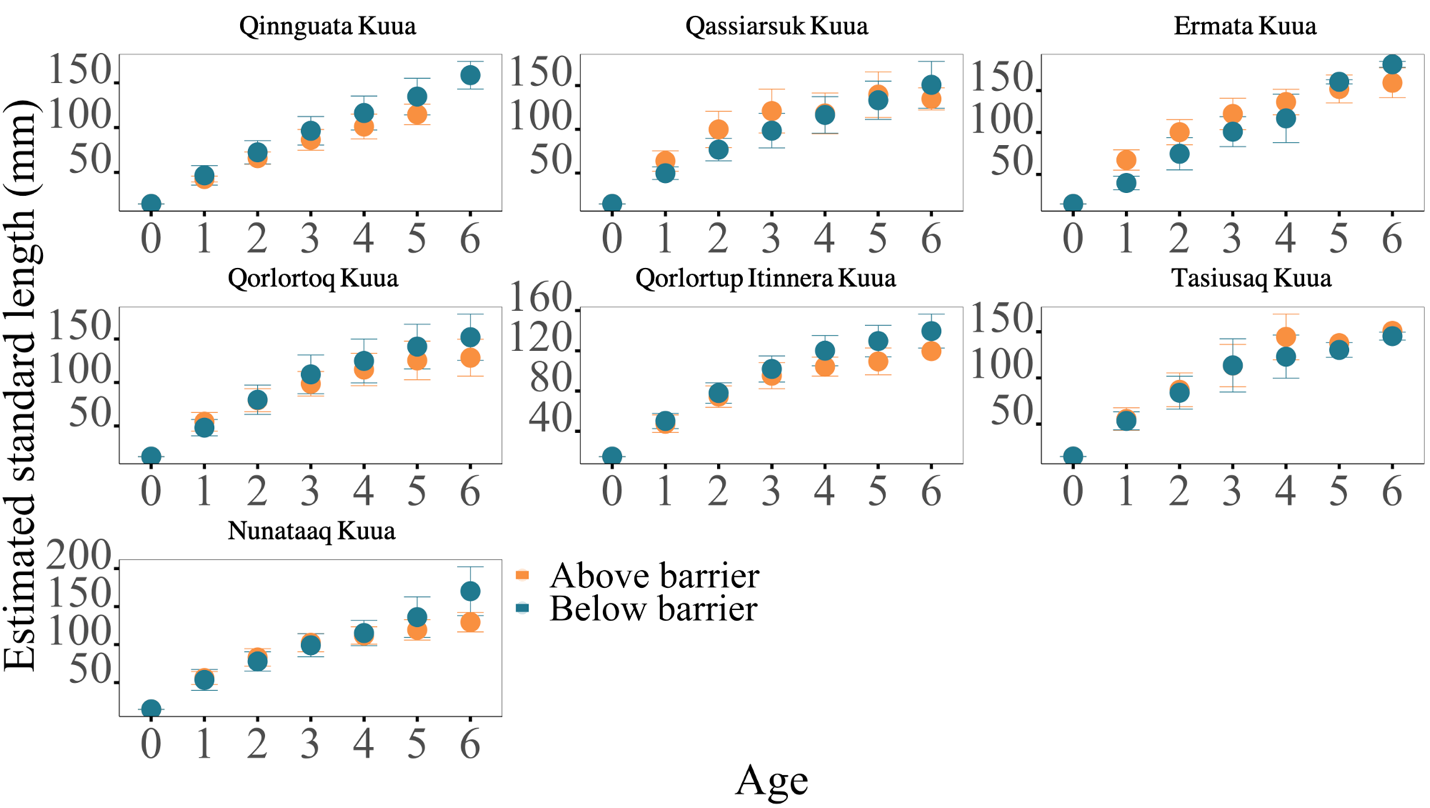
 Figure S14.** Average estimated standard length at age of Arctic charr per stream between above and below barrier populations. Response curves represent predicted values generated from a linear mixed model.
